# Magnetic Iron Oxide Nanoparticles: Advances in Synthesis, Mechanistic Understanding, and Magnetic Property Optimization for Improved Biomedical Performance

**DOI:** 10.3390/nano15191500

**Published:** 2025-10-01

**Authors:** Minh Dang Nguyen, Supawitch Hoijang, Ramtin Yarinia, Melissa Ariza Gonzalez, Suman Mandal, Quoc Minh Tran, Pailinrut Chinwangso, T. Randall Lee

**Affiliations:** 1 Department of Chemistry and the Texas Center for Superconductivity, University of Houston, Houston, TX 77204, USA; 2 School of Materials Science, Indian Association for the Cultivation of Science, Kolkata 700 032, India

**Keywords:** iron oxide nanoparticles, thermal decomposition, iron oleate, iron acetylacetonate, iron pentacarbonyl, spherical nanoparticles, cubic nanoparticles, superparamagnetism, phase transfer

## Abstract

Magnetic iron oxide nanoparticles (MIONPs) represent a versatile magnetic nanoparticle (NP) system with considerable, yet underexplored, potential in diverse applications, particularly in emerging biomedical fields such as magnetic resonance imaging, magnetic hyperthermia, targeted drug delivery, and biosensing. The successful translation of MIONPs into these applications requires reproducible synthesis methods and precise control over particle uniformity in terms of size, shape, and composition. However, reproducibility in nanoparticle synthesis remains a persistent challenge, limiting the ability of researchers to replicate results and integrate MIONPs into application-oriented studies. In recent years, substantial efforts have been directed toward elucidating synthesis mechanisms and improving both reproducibility and particle uniformity, enabling notable advances in the biomedical deployment of MIONPs. This review summarizes progress in the synthesis of MIONPs, with emphasis on three widely employed precursors: iron oleate, iron acetylacetonate, and iron pentacarbonyl. The discussion focuses on key findings in NP synthesis, relevant chemical aspects, and the magnetic properties of MIONPs, which are critical for optimizing their functional performance. By consolidating recent advances, this review aims to provide a reliable framework for the preparation of high-quality MIONPs and to support their effective use in specific biomedical applications.

## 1. Introduction

Magnetic iron oxide nanoparticles (MIONPs) have demonstrated considerable potential for diverse applications in environmental remediation, energy conversion, electronics, sensing, and catalysis. They also hold promise for transforming healthcare through innovative solutions in biomedical and biological applications, spanning both therapeutic and diagnostic domains [1,2]. As the most widely used class of magnetic nanoparticles, commercial MIONPs in the magnetite and maghemite phases are recognized for their biocompatibility and have been approved by the European Medicines Agency (EMA) and the United States Food and Drug Administration (FDA) for various medical uses [3]. Consequently, the development of improved synthesis methods, refinement of established protocols, and production of high-quality nanoparticles (NPs) with tailored magnetic properties have remained central objectives for the research community over the past decades.

Initial research efforts focused on establishing synthetic methods capable of producing NPs with uniform size and shape [1,4]. More recently, attention has shifted toward understanding the synthesis mechanisms, optimizing protocols for improved structural control and reproducibility, and investigating magnetic properties at the microscopic scale. These studies have addressed factors such as crystallographic quality and defects [5], magnetic dead layers [6], exchange bias [7,8,9,10], and even the magnetic behavior of individual NPs [11,12]. It is now recognized that particle size and shape alone do not determine magnetic performance; instead, compositional homogeneity and the nature of crystallographic defects play critical roles. Reproducibility remains a key consideration; a synthesis method capable of producing structurally uniform particles may still yield suboptimal magnetic properties if structural defects are present. Increasingly, studies reveal that nanoscale structural features beyond morphology—including lattice quality and defect distribution—strongly influence magnetic behavior [5,13]. Advancing these understandings will enable more precise control over magnetic properties, enhance performance in biomedical applications, and facilitate the translation of MIONP-based technologies from laboratory research to clinical use.

To date, the criteria for selecting MIONPs for biomedical applications have been shaped by recent developments. Optimized MIONPs should (1) possess appropriate size and desired magnetic properties; (2) exhibit uniformity in size and shape to ensure reproducible performance; (3) minimize crystallographic defects and surface disorders to maximize magnetic properties; and (4) present suitable surface functionalities that ensure biocompatibility and colloidal stability in physiological environments.

The synthesis of MIONPs from three main types of precursors—(1) iron(III) oleate and related salts (e.g., iron(II/III) stearate, iron(II/III) oxalate, iron(III) dodecanoate, iron(III) octanoate); (2) iron(III) acetylacetonate; and (3) iron pentacarbonyl [Fe(CO)_5_]—at elevated temperatures in organic solvents has been demonstrated to be among the most effective and reliable approaches. These methods have attracted considerable attention from chemists and materials scientists aiming to refine and improve the synthesis. In general, they employed either thermal decomposition or solvothermal techniques, which are widely regarded as producing NPs with superior quality compared to other synthetic strategies. A common limitation of these approaches is the generation of hydrophobic nanoparticle surfaces, which restricts their direct use in biomedical applications. However, advances in surface coating and functionalization over the past decades have largely addressed this issue. Current research efforts focus on optimizing chemical synthesis using these three precursor classes, refining protocols, and improving synthetic conditions to achieve NPs with higher crystallinity, enhanced magnetic properties, and improved reproducibility.

Therefore, it is important to summarize recent progress in the synthesis of MIONPs, with primary focus on (1) elucidating the reaction mechanisms and the roles of chemical reagents; (2) improving reproducibility and scalability for the production of high-quality NPs; and (3) identifying synthetic parameters that govern nanocrystal structures and, consequently, the magnetic properties of MIONPs. These advances have been facilitated by the development of advanced characterization techniques in materials science, enabling detailed analysis of structure, composition, and properties at the microscopic level, as well as real-time monitoring of nanocrystal nucleation and growth.

In comparison with other reviews that aim to comprehensively cover content or focus on specific applications, we selectively summarize recent advances that best illustrate the main theme of our paper and articulate its three primary focuses. For a comprehensive understanding of MIONPs or magnetic NPs, ranging from available synthesis methods to diverse applications, the reviews by the Yadav group [14], the Liu and Sun group [15], and the Hyeon group [16,17,18,19] are highly recommended. In addition, several reviews dedicated to specific topics provide valuable insights, including studies on the relationship between size, shape, and crystallinity of MIONPs and their effects on magnetic properties [1]; multifunctional IONPs for biomedical applications [2]; exchange bias in MIONPs [20]; magnetic hyperthermia application [21]; MIONPs for brain imaging and drug delivery [22]; surface modifications of MIONPs [23]; nanogels for combined hyperthermia and drug delivery for cancer treatment [24]; and applications and potential toxicity of MIONPs [25].

**Scheme 1:**
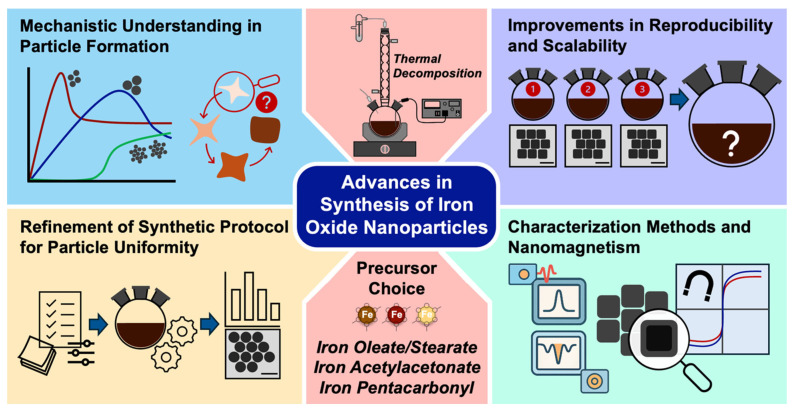
Key Research Focus Areas in the Chemical Synthesis of Magnetic Iron Oxide Nanoparticles (MIONPs).

Scheme 1 illustrates the scope of this review, which centers on synthetic methods employing thermal decomposition or solvothermal reactions of organometallic complexes—such as iron(III) oleate, iron(III) stearate, iron(III) acetylacetonate, and iron pentacarbonyl—in organic solvents at elevated temperatures. The review focuses on chemical synthesis strategies and the magnetic properties of the resulting NPs, providing both a concise reference for researchers developing synthetic methodologies and guidance for scientists designing applications of magnetic NPs to select methods that best align with their objectives. Our discussion begins with an overview of synthetic approaches, followed by an examination of characterization techniques used to establish structure–property relationships. Recent key findings in understanding and optimizing the magnetic properties of MIONPs are also highlighted. Finally, a brief discussion addresses phase-transfer strategies for converting hydrophobic MIONPs obtained from thermal decomposition into hydrophilic counterparts suitable for biomedical applications.

## 2. Synthesis of Magnetic Iron Oxide Nanoparticles

### 2.1. Iron Oleate, Iron Stearate, and Related Precursors

***Establishing Iron Oleate as a Key Precursor in Iron Oxide Nanoparticle Synthesis.*** Iron(III) oleate (FeOL) was first identified as an effective precursor for the synthesis of iron oxide nanoparticles (IONPs) by the research groups of Peng and Hyeon in 2004 [26,27]. Initially, FeOL was discovered as an in situ intermediate during the thermal decomposition of Fe(CO)_5_ in the presence of oleic acid (OA), where it acted as an efficient growth source for monodisperse IONPs [27,28]. FeOL is typically prepared by refluxing iron(III) chloride (FeCl_3_) with sodium oleate (NaOL) in a ternary solvent mixture of ethanol, water, and hexane at 70 °C, producing a waxy black-brown solid. Compared to Fe(CO)_5_, metal chlorides and NaOL are more cost-effective, less toxic, and environmentally friendly.

Highly monodisperse, superparamagnetic iron oxide (IO) nanospheres have been obtained via thermal decomposition (“heating-up” process) of in-house-prepared FeOL in the presence of OA at 320 °C [27]. Mechanistic insights from transmission electron microscopy (TEM), thermogravimetric analysis (TGA), differential scanning calorimetry (DSC), and in situ temperature-programmed infrared spectroscopy revealed that nucleation occurs at 200–240 °C via dissociation of one oleate ligand from Fe(oleate)_3_ through CO_2_ elimination. Major crystal growth proceeds at ~300 °C, driven by removal of the remaining two oleate ligands. Particle diameters between 5 and 16 nm were achieved using various high-boiling-point organic solvents, including 1-hexadecene (b.p. 274 °C), octyl ether (b.p. 287 °C), 1-octadecene (ODE; b.p. 317 °C), 1-eicosene (b.p. 330 °C), and trioctylamine (TOA; b.p. 365 °C) [27]. Solvents with higher boiling points promoted larger particle formation due to enhanced FeOL reactivity. Particle size could also be tuned by adjusting the OA concentration.

X-ray absorption spectroscopy (XAS) and X-ray magnetic circular dichroism (XMCD) indicated that 5 nm IONPs were predominantly maghemite (γ-Fe_2_O_3_, Fe^3+^ only), whereas magnetite (Fe_3_O_4_) content increased with particle size. This method yielded up to 40 g of high-quality IONPs, demonstrating industrial scalability. Furthermore, Hyeon et al. showed that nanoparticle formation in the heating-up method follows the classical LaMer model [29,30]. Since its introduction, FeOL has remained a central precursor for IONP synthesis, inspiring numerous advances over the past two decades.

**Figure 1:**
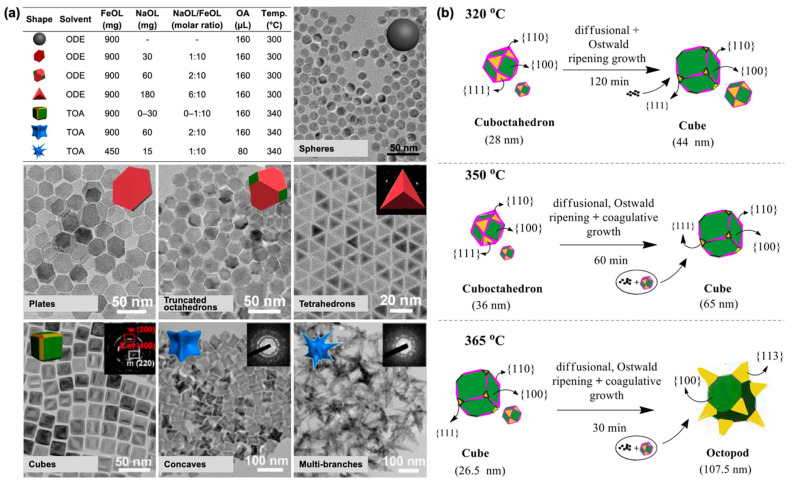
(**a**) Reaction conditions and corresponding TEM images for the synthesis of IONPs with various shapes and surface structures, including isotropically grown IO spheres; {111} facet-exposed IO plates, truncated octahedrons, and tetrahedrons; and {100} facet-exposed IO cubes, concaves, and multi-branched structures. Reproduced with permission from Ref [31]. Copyright 2015 American Chemical Society. (**b**) Schematic illustration of the proposed growth mechanisms of anisotropic wüstite (FeO)−spinel (magnetite, Fe_3_O_4_/maghemite, γ-Fe_2_O_3_) core−shell IONPs at various reaction temperatures and aging times. Reprinted with permission from Ref. [32]. Copyright 2020 American Chemical Society.

***Iron Oleate as a Versatile Precursor for Morphology-Controlled Iron Oxide Nanoparticles.*** In addition to spherical shape, other well-defined morphologies of IONPs can be obtained by optimizing the thermal decomposition of FeOL. Gao et al. developed a synthesis protocol capable of producing a diverse range of morphologies and surface structures [31]. By varying the reaction temperature, employing two different solvents—ODE and TOA—and tuning the molar ratio of NaOL to FeOL, various IONP morphologies with distinct facet exposures were synthesized, as illustrated in Figure 1a. Using ODE (a lower-boiling-temperature solvent), IONPs predominantly exposing {111} facets, including hexagonal plates, truncated octahedrons, and tetrahedrons, were obtained. NaOL plays a critical role in modulating the surface energy of the {111} facet by preferentially binding free ionic oleate (OL^−^) ligands to high-density iron ions, as the {111} facet exhibits both a higher density of iron ions and lower attachment energy compared with other facets [31,32,33]. Consequently, growth proceeded along other facets, preserving the exposure of {111} facets in the final IO products.

In contrast, in TOA (a higher-boiling-temperature solvent), cubic IONPs were obtained even without the initial addition of NaOL, due to the in situ generation of NaOL through deprotonation of OA by TOA. Increasing the NaOL content led to an evolution from cubic to concave-like shapes, resulting from epitaxial growth, and subsequently to multi-branched structures, attributed to delayed nucleation kinetics. All structures synthesized in the TOA system typically exhibited a core–shell wüstite (FeO)@magnetite (Fe_3_O_4_) architecture, due to the rapid growth rate at elevated temperatures exceeding the oxidation rate from FeO to Fe_3_O_4_ [31,32,34,35]. Building on these findings, a recent study demonstrated that concave (or octapod) IONPs with diameters of 25–50 nm were successfully synthesized under comparable conditions using FeOL in TOA with NaOL and OA [35]. The magnetic reversal process of these anisotropic IONPs was also investigated to gain deeper insight into the magnetization reversal mechanism, providing valuable guidance for their performance in biomedical applications such as magnetic resonance imaging (MRI) contrast agent and magnetic hyperthermia [35].

The solvent serves as a medium to dissolve reagents and facilitate nanoparticle growth; however, in many cases, it also acts as a coordinating agent that can influence particle morphology. For example, varying the solvent composition from 100% TOA (a coordinating solvent) to 100% ODE (a non-coordinating solvent) enabled tuning of IONP morphology from anisotropic concave nanocubes to isotropic nanospheres [34]. This transition was attributed to TOA’s preferential binding to low-index facets such as {100}, where its bulky structure sterically hindered growth and promoted the formation of high-index faceted concave structures. Regardless of the resulting morphology, all as-synthesized IONPs initially exhibited the wüstite phase. The bulkiness of the coordinating solvent is an important factor. For example, the use of a primary amine such as OAm, which has a boiling point similar to that of TOA (approximately 364 °C), resulted in the formation of anisotropic but irregular shapes [34]. This observation highlights the crucial role of bulky tertiary amine molecules in directing the formation of concave nanocube IONPs. Mechanistic insights into the formation of anisotropic IONPs with cuboctahedral, cubic, and octopod morphologies—each displaying core–shell architectures—were provided in a recent study by Ravikumar’s group [32]. These structures were obtained via the thermal decomposition of FeOL in TOA with OA by varying the reaction time (aging) at 320, 350, and 365 °C. Based on experimental observations, particle growth at 320 °C proceeded predominantly via diffusion-controlled and Ostwald ripening mechanisms [30]. At 350 and 365 °C, Brownian coagulation also contributed to growth, in addition to diffusion-controlled and Ostwald ripening mechanisms, as illustrated in Figure 1b. The formation of these distinct morphologies was dictated by the competition between the monomer deposition rate onto {111} facets and surface diffusion toward {100} facets, as described by a timescale-based particle growth model.

The role of OA and its quantity in the formation pathway of IONPs was recently investigated by Solodov et al. [36]. Nuclear magnetic resonance (NMR) relaxation measurements were used to study the nucleation and growth mechanisms of IONPs synthesized via a heating-up method from an FeOL precursor [36]. NMR relaxation enabled in situ monitoring of IONP formation by tracking changes in the *T*_1_ (spin–lattice relaxation) to *T*_2_ (spin–spin relaxation) ratio, which correlated with particle size. This approach allowed discrimination between continuous and burst nucleation pathways, depending on the presence of OA. In the absence of OA, nucleation and growth occurred continuously. In contrast, when excess OA was present, the process followed the classical LaMer “burst nucleation” model, with temporally separated nucleation and growth stages [30,36]. OA delayed nucleation by dissolving nascent nuclei and facilitated sharp supersaturation, chemically mimicking the “hot injection” approach, in which precursors are rapidly introduced into a hot solvent to induce instantaneous nucleation. These findings highlight OA’s pivotal role in controlling nucleation dynamics and achieving uniform particle size distribution.

In addition to OA’s role, the introduction of chloride (Cl^−^) ions into the thermal decomposition of FeOL in ODE with OA enabled the formation of size-tunable IONPs with octopod morphology, as Cl^−^ ions selectively bound to iron ions exposed on high-index facets [37]. The resulting octopod IONPs, with an average edge length of 30 nm, exhibited exceptionally high *T*_2_ relaxivity, making them promising candidates as *T*_2_ MRI contrast agents.

**Figure 2:**
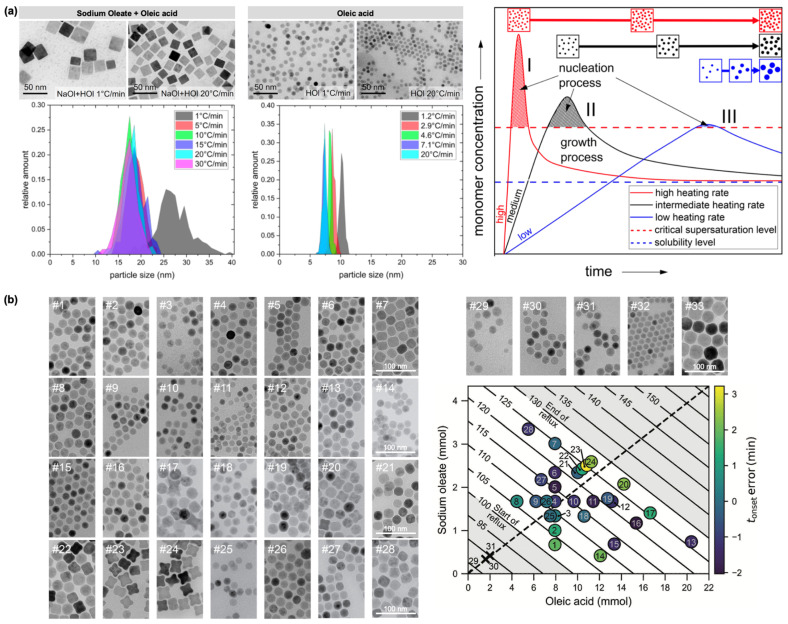
(**a**) TEM images and corresponding size distributions of IONPs synthesized in the presence of NaOL/OA or OA alone under different heating rates. Also shown is a LaMer model diagram illustrating the nucleation and growth process during IONP formation as influenced by heating rate. Reprinted with permission from Ref. [38]. Copyright 2022 Springer Nature. (**b**) TEM images of 33 batches of IONPs synthesized by varying the solvent and the amounts of OA and NaOL surfactants, together with a corresponding 2D plot illustrating the onset of burst nucleation. Reprinted with permission from Ref. [40]. Copyright 2024 John Wiley & Sons.

The heating rate during FeOL thermolysis also significantly affects IONP size. Thermal decomposition of FeOL in the presence of NaOL and OA showed a decrease in the average edge length of cubic IONPs with increasing heating rate (Figure 2a) [38]. A similar trend was observed for spherical IONPs synthesized with OA alone. The largest particles were obtained at the lowest heating rate (1 °C·min^−1^), whereas higher heating rates (>5 °C·min^−1^) yielded NPs with relatively uniform sizes, likely due to precursor depletion during the nucleation phase. Different surfactants produced distinct morphologies: NaOL/OA mixtures yielded cubic NPs, while OA alone produced spherical ones, reflecting their differential stabilizing effects on the precursor and facet-selective adhesion.

The LaMer model explains this heating rate dependence: high heating rates generate strong supersaturation, producing numerous nuclei and smaller particles, whereas low heating rates result in mild supersaturation, fewer nuclei, and thus larger particles (Figure 2a) [30,38]. Most synthesized NPs exhibited a core–shell structure, with a wüstite (FeO) core surrounded by a spinel shell (γ-Fe_2_O_3_ or Fe_3_O_4_). Consistent with these observations, a factorial design-of-experiments study—a systematic optimization approach that evaluates multiple synthesis parameters and their interactions to enable reliable and reproducible protocols—demonstrated that high heating rates promote burst nucleation, yielding highly monodisperse 12–14 nm IONPs with strong magnetization, whereas low heating rates lead to gradual nucleation, larger 22–24 nm IONPs, broader or multimodal size distributions, and reduced magnetization, likely due to Ostwald ripening and possible FeO formation [39].

A recent study reported that changes in reflux temperature can serve as an indicator of IONP formation [40]. This phenomenon was characterized by a reproducible temperature profile, consisting of a small endothermic dip (ΔT ≈ −3 °C) followed by a slight increase (ΔT ≈ +3 °C), marking the onset of nucleation. This temperature marker revealed a linear relationship between surfactant concentration (OA and NaOL) and nucleation onset (Figure 2b), supporting a chemically activated burst nucleation mechanism. Exploiting this correlation, highly uniform IONPs with precisely controlled morphologies—spherical, cubic, and star-shaped—ranging from 12 to 30 nm were synthesized (Figure 2b). This finding not only simplified reaction monitoring and improved reproducibility but also provided practical guidelines for rapidly achieving targeted nanoparticle shapes and sizes.

Despite these advances in morphology control via thermal decomposition, challenges remain due to reproducibility and structural consistency issues of the FeOL precursor.

***Advances and Challenges in the Preparation of Iron Oleate Precursors for Controlled Nanoparticle Synthesis.*** Despite its popularity and versatility in nanoparticle synthesis, FeOL presents significant challenges in reproducibility and structural consistency. The widely used FeOL precursor is typically prepared in-house due to the lack of commercial availability. Common issues associated with FeOL synthesized by reacting iron(III) salts with OA include (1) non-stoichiometric composition, attributed to the various coordination modes by which long-chain carboxylates bind to iron ions; (2) difficulty in purification using standard techniques such as recrystallization or column chromatography; and (3) batch-to-batch variability [41]. Substantial evidence indicates that FeOL compounds are structurally diverse, with their ligation, nuclearity, solvation, and oxidation state highly sensitive to synthetic conditions [41,42]. These factors undermine sample-to-sample consistency and hinder predictive, scalable control over nanoparticle size, dispersity, morphology, and phase.

**Figure 3:**
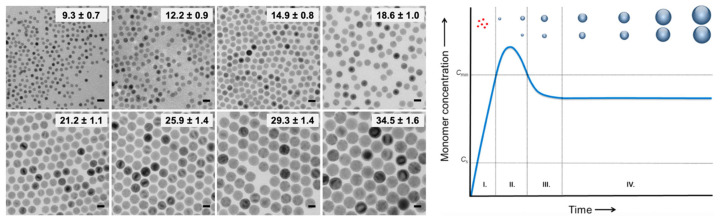
TEM images of IONPs of various sizes synthesized using the extended LaMer approach (scale bars: 20 nm), together with a schematic illustration of the proposed extended LaMer mechanism for nanoparticle growth, enabling precise size control. Reproduced with permission from Ref. [43]. Copyright 2015 American Chemical Society.

A new approach, termed the “extended LaMer” mechanism, was introduced to address these limitations, enabling the synthesis of uniform IONPs with tunable size via continuous precursor addition [43]. In this study, the FeOL—used without purification—was prepared by thermally decomposing iron acetylacetonate (Fe(acac)_3_) in neat OA at 290 °C. Continuous addition of diluted FeOL in ODE at a constant rate produced highly crystalline, uniform IONPs ranging from 9.3 to 34.5 nm in diameter (Figure 3). Continuous precursor addition maintained monomer generation until the rate of monomer consumption by growing NPs equaled the rate of monomer production, resulting in a steady-state monomer concentration. This modified the classic LaMer mechanism by introducing a new Stage IV, representing steady-state growth, as schematically illustrated in Figure 3 [30,43]. Stage IV can be prolonged arbitrarily, enabling the synthesis of particles across a broad size range with low dispersity. Maintaining a constant growth rate during State IV ensured reproducibility and predictability while preventing Ostwald ripening and secondary nucleation events.

Using the same method for synthesizing IONPs, a study on the effects of addition rate and reaction time revealed that reaction time had a greater impact on particle size and magnetic saturation than addition rate at moderate speeds [44]. Similarly, a continuous flow approach—in which a mixture of precursor, surfactant, and solvent passes through a heated coil (microreactor) at controlled temperature—was successfully developed to achieve size-tunable IONPs [45]. Under optimized conditions, this flow microreactor presents a promising strategy for the scalable production of uniform IONPs.

The drying period of the FeOL precursor after preparation reliably influenced the particle size of IONPs [46,47]. For example, thermal decomposition of FeOL dried at 30 °C for durations ranging from 5 to 30 days produced spherical IONPs with diameters between 6.3 and 12.8 nm and high monodispersity (polydispersity < 5%) [46]. The effect of drying temperature was also investigated by comparing FeOL dried at 60 °C; no significant difference in particle size or uniformity was observed once sufficient drying time was allowed. Furthermore, IONPs synthesized from two independent FeOL batches under identical drying conditions exhibited consistent average particle sizes within 0.1–0.3 nm [46]. Variation in drying time altered the coordination environment of Fe centers, thereby tuning the nucleation-to-growth ratio of iron species and enabling precise size control with high monodispersity in the resulting IONPs.

In addition to drying time, the initial molar ratio of Fe(III) to NaOL in FeOL preparation affected the size, shape, and uniformity of IONPs [47]. An Fe(III)-to-NaOL molar ratio near or below 1:3 favored the formation of well-defined octahedral NPs with narrow size distributions, whereas a higher NaOL excess led to more faceted or less uniform morphologies, such as cuboctahedra. This effect arises from changes in the coordination environment and stoichiometry of FeOL complexes, which influence nucleation and growth behavior during thermal decomposition.

**Figure 4:**
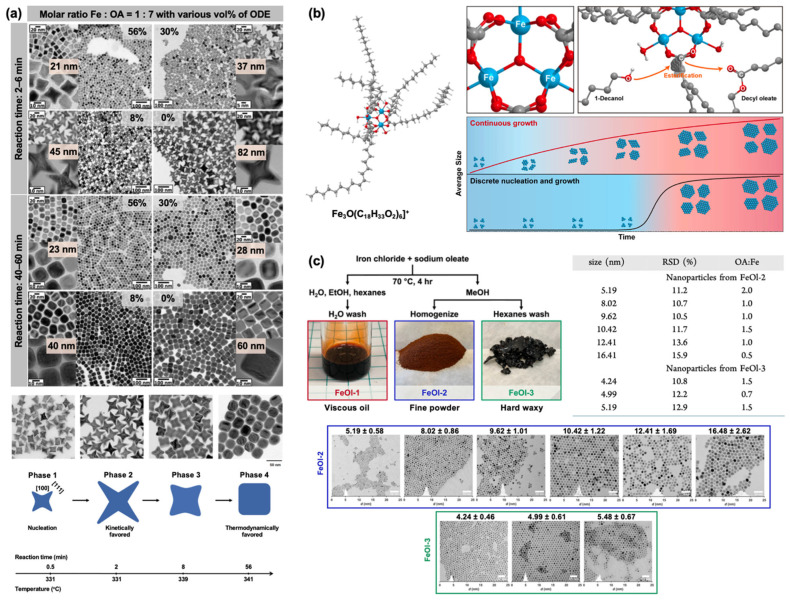
(**a**) TEM images of IONPs exhibiting star-like and cubic morphologies corresponding to 2–6 min and 40–60 min after nucleation, respectively, under varying concentrations of FeOL diluted in ODE. Also shown are TEM images illustrating particle size and shape evolution over a 1 h reaction period, accompanied by a schematic representation of the distinct developmental stages (Fe/OA = 1:7; ODE = 8 vol%). Reprinted with permission from Ref. [33]. Copyright 2019 American Chemical Society. (**b**) Computed structure of the trinuclear-oxo carboxylate complex of FeOL ([Fe_3_O(C_18_H_33_O_2_)_6_]^+^) with a magnified view of its core structure; proposed continuous growth mechanism driven by esterification; and schematic comparison between the continuous growth model and the conventional discrete nucleation–growth pathway. Reprinted with permission from Ref. [48]. Copyright 2019 American Chemical Society. (**c**) Synthetic scheme and representative photographs of FeOL precursors prepared via a modified conventional method, together with a table presenting the size and dispersity of the obtained IONPs and their corresponding TEM images. Reprinted with permission from Ref. [49]. Copyright 2022 American Chemical Society.

A new preparation route for FeOL precursors was developed using iron(II) carbonate (FeCO_3_) and iron(III) carbonate (Fe_2_(CO_3_)_3_) as iron sources to achieve high-purity iron oleate and to separate the oleate formation step from nanoparticle nucleation and growth, thereby enabling precise control over the reaction mechanism and resulting nanocrystal properties [33]. The resulting iron(II) and iron(III) oleate complexes were subsequently decomposed thermally to form IONPs. Regardless of the initial oxidation state of the carbonate precursor, IONPs with identical shape, size, and crystal structure were obtained, indicating a common reaction pathway. Systematic variation of reaction time and FeOL concentration (diluted with ODE, vol%) revealed that star-shaped (octapod) IONPs formed as kinetically favored products only during the early growth stage (2–6 min), eventually evolving into cube-like structures (Figure 4a). The largest octapods were obtained in pure OA (0 vol% ODE), whereas moderate dilution (8 vol% ODE) significantly reduced particle size. A four-phase shape evolution mechanism was proposed: (1) nucleation and growth of small cubes with {100} and {111} facets; (2) OA-stabilized {100} facets promoting anisotropic growth along <111> directions, forming octapods within ~2 min; (3) surface diffusion (~8 min) smoothing octapods into truncated cubes; and (4) Ostwald ripening, producing larger cubes at the expense of smaller ones [30]. This approach yielded up to 20 g per batch, demonstrating scalability. The as-prepared IONPs exhibited a core–shell-like morphology after ambient storage, with a wüstite core and a maghemite/magnetite-rich shell.

Identification of the FeOL molecular structure is essential for understanding IONP formation mechanisms. Park and Hyeon’s group employed multiple characterization techniques—including matrix-assisted laser desorption/ionization time-of-flight mass spectrometry (MALDI-TOF MS), Fourier transform infrared (FTIR), and near-infrared (NIR) spectroscopy—to analyze the chemical structure of FeOL prepared via the conventional reaction of FeCl_3_ with NaOL [48]. Results indicated that FeOL was not a mononuclear complex but a trinuclear iron-oxo carboxylate cluster with *μ*_3_-oxygen bridges (Figure 4b). Thermal decomposition of the FeOL in 1-decanol, serving as both solvent and reaction promoter, was studied using NMR, mass spectrometry, TEM, and in situ X-ray scattering. A “continuous growth mechanism” was proposed, in which esterification of bound carboxylates generated hydroxyl groups on trinuclear clusters, enabling condensation into larger iron-oxygen intermediates (e.g., Fe_2_, Fe_4_, Fe_5_, and Fe_6_) that progressively grew into NPs. This mechanism, analogous to nonhydrolytic sol–gel chemistry, involved no distinct nucleation stage, likely due to the stability of intermediate clusters maintained by strong carboxylate binding. This study clarified FeOL structure and introduced a mechanistic framework distinct from the classical nucleation model.

Recently, two new stoichiometric FeOL forms—fine powder and hard wax—were prepared via a modified synthesis route (Figure 4c) [49]. Thermal decomposition of the powder form under varying Fe-to-OA ratios and solvent contents produced spherical IONPs (5–16 nm), whereas the wax form yielded monodisperse 4–5 nm IONPs. Multiple synthesis batches demonstrated consistent size and uniformity. These precursors were isolable in bulk, stable during storage, and chemically well-defined, enabling predictable reactivity. Incorporation of other divalent metal salts into the FeOL synthesis route yielded mixed-metal oleates, suitable for producing various spinel ferrites (*M*Fe_2_O_4_, where *M* represents divalent metals such as Mn, Co, Ni, and Cu) [50].

***Strategies for Synthesizing Phase-Pure Iron Oxide Nanoparticles: Overcoming Wüstite Formation in FeOL-Based Systems.*** Phase compositional control of IONPs synthesized from FeOL precursors is a critical issue, as it directly determines their magnetic properties and application-specific performance. IONPs typically occur in three magnetic phases: fully oxidized maghemite (γ-Fe_2_O_3_), mixed-valent magnetite (Fe_3_O_4_), and metastable reduced wüstite (FeO). Magnetite and maghemite adopt an inverse spinel ferrimagnetic (FiM) configuration, whereas wüstite is antiferromagnetic (AFM) below its Néel temperature (~190 K). The presence of wüstite generally diminishes magnetic performance due to its AFM contribution. Although recent studies have shown that exchange bias between an FeO AFM core and an Fe_3_O_4_ FiM shell can modulate anisotropy for biomedical applications, minimizing FeO formation remains a primary objective. A widely applied strategy to remove AFM wüstite is post-synthetic oxidation, typically achieved by thermal annealing in organic solvents under air or in the presence of oxidizing agents such as trimethylamine *N*-oxide [34,40,51,52,53]. In addition, in situ oxidation approaches have been developed to favor the direct formation of phase-pure FiM IONPs, thereby eliminating the need for post-synthetic treatments.

For example, IONPs synthesized by thermal decomposition of FeOL in octadecane, ODE, squalene (SQE), and dioctyl ether exhibited core–shell architectures, except for those synthesized in dibenzyl ether (DBE) [52]. Thermolysis of DBE produces free radicals that act as in situ oxidizing agents, preventing wüstite formation. Moreover, blending DBE with other solvents to raise the reaction temperature above 300 °C enabled the synthesis of nearly phase-pure IONPs with tunable sizes (10–30 nm) and magnetic properties ranging from superparamagnetic to ferromagnetic. This finding highlighted the solvent crucial role in controlling phase composition.

Gas atmosphere also strongly affects phase purity. An in situ oxidation method employing 1% oxygen (O_2_) in argon (Ar) promoted the formation of phase-pure magnetite [54]. This O_2_/Ar mixture was suitable for scale-up, as the O_2_ concentration was below the limiting oxygen concentration (LOC) required to sustain combustion. While effective, this approach required more than 30 h for larger particles and necessitated size-dependent optimization. To improve efficiency, thermal decomposition of FeOL by both conventional “heating-up” and the “Extended LaMer” methods was optimized using molecular oxygen (20% O_2_/Ar) [6]. In the presence of O_2_, uniformly single-crystalline IONPs were obtained, unlike those produced under inert conditions. Importantly, the physical diameters (measured by TEM) closely matched the magnetic diameters (determined by magnetic characterization), indicating reduced structural defects and minimized magnetically dead layer. Consistent O_2_ feeding across multiple batches further improved reproducibility. Similarly, flowing 5% O_2_ during reflux was shown to maintain an oxidizing environment and yielded phase-pure magnetite [49].

Lower-temperature esterification-mediated synthesis (~230 °C) has also been reported to suppress FeO formation [55]. In this approach, esterification between OA and oleyl alcohol generated reactive iron hydroxyl species without requiring high-temperature thermal decomposition. This reduced the extent of iron(III) reduction to iron(II), thereby favoring spinel-phase growth under milder, less reducing conditions.

**Figure 5:**
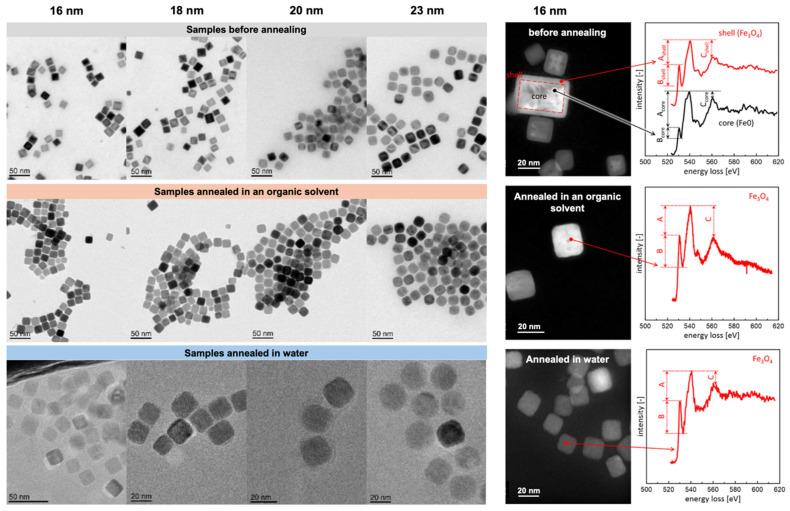
(**left**) TEM images of FeO@Fe_3_O_4_ nanocubes with different edge lengths, shown as-synthesized (without thermal treatment) and after annealing in either an organic solvent or water. (**right**) Scanning TEM images with corresponding EELS spectra of 16 nm nanocubes before and after annealing in an organic solvent and water. Reprinted with permission from Ref. [10]. Copyright 2023 American Chemical Society.

A recent comparative study investigated thermal annealing of FeO@Fe_3_O_4_ nanocubes (edge lengths: 16–23 nm) in both ODE (organic media) and water (aqueous media) [10]. A water transfer step was necessary prior to aqueous annealing. As shown in Figure 5, the morphology of nanocubes remained unchanged after both treatments, while the core–shell architecture transformed into single-phase magnetite. Notably, nanocubes smaller than 20 nm annealed in organic solvent exhibited stronger magnetic particle imaging (MPI) signals than those annealed in aqueous media.

Despite these advances in FeOL-based systems, alternative iron precursors are increasingly being explored to further improve synthesis control, reproducibility, and phase selectivity.

***Iron Stearate and Related Compounds as Alternatives to Iron Oleate for Controlled Synthesis of Iron Oxide Nanoparticles.*** In addition to FeOL, iron(II) and iron(III) stearates (FeSt) have been employed as precursors for IONP synthesis via thermal decomposition. Like FeOL, FeSt enables the formation of IONPs with diverse morphologies and high uniformity [56,57]. FeSt is commercially available, exhibits greater stability against aging and hydration, and may improve reproducibility [56,58]. Bégin-Colin et al. demonstrated that variations in the chemical structure of FeSt precursors—specifically the number of ligand chains and the degree of hydration—strongly influenced IONP morphology [59,60]. Iron(II) stearate (FeSt_2_), with two stearate chains, and iron(III) stearate (FeSt_3_), with three, were studied in both hydrated and dehydrated forms. FeSt_2_ preferentially yielded nanoplates, whereas FeSt_3_ favored nanocubes [60]. Hydration hindered anisotropic growth by promoting ligand–water interactions that facilitated micelle formation, while dehydrated precursors allowed greater morphological control [59,60].

Structural analyses of FeSt_2_ and FeSt_3_ prepared by reacting FeCl_2_ and FeCl_3_ with sodium stearate revealed polynuclear complexes [61]. FeSt_2_ primarily contained [Fe_3_(*μ*_3_-O)St_6_·*x*H_2_O]Cl, whereas FeSt_3_ consisted of [Fe_3_(*μ*_3_-O)St_6_·*x*H_2_O]St, larger complexes such as [Fe_7_(*μ*_3_-O(H))_6_(*μ*_2_-O(H))*ₓ*St_12−2*x*_], and free stearic acid. The larger, thermally stable complexes in FeSt_3_ were attributed to higher hydrolysis and condensation rates in iron(III) solutions. Nucleation was proposed to proceed via condensation of iron(III)-based polycations into FeO nuclei. These stable complexes hindered complete decomposition at lower temperatures, affecting nanoparticle growth.

Further mechanistic insights into the nucleation mechanism of IONPs derived from FeSt_2_ revealed a confinement-driven process [62]. Polynuclear complexes became trapped in micelle-like reverse aggregates formed by OA in the organic solvent, where confinement accelerated condensation and promoted FeO nucleation. This study emphasized that precursor structure and confinement within the organic phase, rather than bulk thermal stability alone, play decisive role in controlling nucleation, particle size, and monodispersity.

A recent comparison between in-house prepared and commercial FeSt_3_ highlighted precursor-dependent reproducibility [5]. Under identical conditions, in-house FeSt_3_ yielded spherical IONPs with broad size variation (16–27 nm), whereas the commercial precursor produced narrower size distributions (23–26 nm) with fewer structural defects and more uniform phase formation. Characterization revealed differences in carboxylate coordination, polycation species, and thermal stability, confirming that synthesis of FeSt_3_ is particularly sensitive due to its tendency to form larger, less consistent polycations.

Rational precursor design has further advanced phase control. For example, the heterometallic μ_3_-oxo cluster [Fe^2+^Fe_2_^3+^O(O_2_CCF_3_)_6_(H_2_O)_3_] was employed as a single-source precursor to synthesize stoichiometric, monodisperse IONPs [63]. Thermal decomposition in oleylamine (OAm) at 300 °C produced highly crystalline IONPs (~5.6 nm) with the expected mixed-valent Fe^2+^/Fe^3+^ ratio and inverse spinel magnetite structure. In contrast, decomposition of conventional Fe^3+^—only oxo clusters, such as [Fe_3_O(C_18_H_33_O_2_)_6_]^+^, yielded wüstite under similar conditions [48]. These findings demonstrated that molecular-level control of oxidation state and stoichiometry enables targeted phase formation, offering improved reproducibility over traditional multi-component methods.

**Figure 6:**
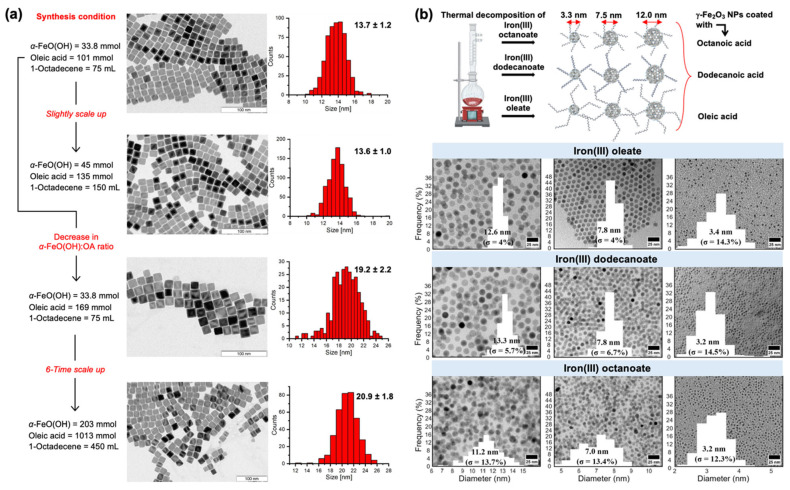
(**a**) TEM images and corresponding size histograms of iron oxide nanocubes synthesized by thermal decomposition of *α*-FeO(OH) under different conditions. Reprinted with permission from Ref. [65]. Copyright 2021 American Chemical Society. (**b**) TEM images of γ-Fe_2_O_3_ NPs synthesized using iron(III) oleate, iron(III) dodecanoate, and iron(III) octanoate as precursors under optimized conditions. Reprinted with permission from Ref. [66]. Copyright 2024 American Chemical Society.

Other simple iron precursors have also proven effective. Iron(II) oxalate (Fe(ox)) enabled size-tunable IONP synthesis through stepwise thermal decomposition [64]. Monodisperse seeds were first generated, followed by seed-mediated growth in OAm and *N*,*N*-diethyl-1,3-diaminopropane (dedap). Coordination of dedap to Fe(ox) lowered the decomposition temperature and enabled size control by varying the amount of precursor decomposed within a fixed solvent volume. Similarly, geothite (*α*-FeO(OH)) was used to synthesize uniform nanocubes (Figure 6a) [65]. The method was scalable, yielding up to 13 g per batch without loss of morphology.

Recently, iron(III) oleate (C18), iron(III) dodecanoate (C12), and iron(III) octanoate (C8) were synthesized and used to prepare spherical γ-Fe_2_O_3_ NPs via thermal decomposition in the presence of corresponding fatty acid surfactants [66]. Depending on ligand length and decomposition behavior, γ-Fe_2_O_3_ NPs with diameters of 3–12 nm were obtained with a low size polydispersity (Figure 6b). This work represented the first example of size-controlled, low-dispersity γ-Fe_2_O_3_ NPs produced by thermal decomposition with tailored surface chemistry. Nonetheless, for many applications, subsequent surface modification remains essential to achieve aqueous dispersibility.

### 2.2. Iron(III) Acetylacetonate Precursor

***Establishing Iron(III) Acetylacetonate as a Key Precursor for Iron Oxide Nanoparticle Synthesis.*** Thermal decomposition of iron(III) acetylacetonate (Fe(acac)_3_) has attracted considerable attention as a route to IONPs, offering several advantages over other precursors. These include commercial availability, ambient stability, tunable size and shape control, and access to highly pure magnetite phases. Unlike iron oleate, which requires in situ preparation, and Fe(CO)_5_, which is highly air-sensitive, Fe(acac)_3_ is stable and convenient for laboratory use. Depending on reaction parameters, Fe(acac)_3_ decomposition yields spherical, cubic, octahedral, and star-like morphologies [67,68,69,70]. Solvents such as benzyl ether (BE) and phenyl ether are commonly employed to promote nucleation and, in some cases, to direct shape evolution. Upon decomposition in the presence of OA, Fe(acac)_3_ readily forms iron oleate intermediates that mediate crystal growth [71,72,73,74,75]. A limitation of this system is the frequent reliance on BE, which is thermally unstable under reaction conditions and often compromises reproducibility. Initially, Fe(acac)_3_ was recognized for producing spherical superparamagnetic IONPs; subsequent studies have extended its application to ferromagnetic nanocubes across broad size ranges. While the primary focus of this paper is on SPIONs, the synthesis of ferromagnetic Fe_3_O_4_ nanocubes also provides important mechanistic insights.

**Figure 7:**
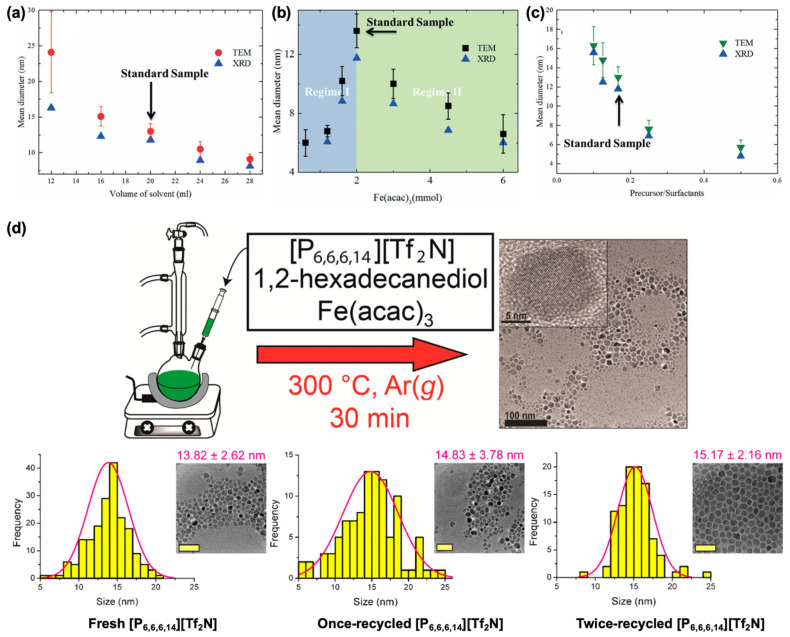
Size evaluation of IONPs as a function of (**a**) solvent volume, (**b**) precursor amount, and (**c**) precursor-to-surfactant ratio. Reproduced with permission from Ref. [79]. Copyright 2017 The Royal Society of Chemistry. (**d**) Thermal decomposition of Fe(acac)_3_ within the ionic liquid trihexyltetradecylphosphonium bis(trifluoromethylsulfonyl)imide ([P_6,6,6,14_][Tf_2_N]) using 1,2-hexadecanediol as a polyol reducing agent and representative TEM images of IONPs synthesized using fresh and recycled [P_6,6,6,14_][Tf_2_N] (scale bar: 50 nm). Reproduced with permission from Ref. [90]. Copyright 2017 American Chemical Society.

The use of Fe(acac)_3_ for IONP synthesis was first introduced by Sun et al. [76] In this method, Fe(acac)_3_ was dissolved in phenyl ether with 1,2-hexadecanediol (HDD) as a reducing agent and OA and OAm as surfactants. HDD partially reduced Fe^3+^ to Fe^2+^, yielding mixed-valent Fe^2+^/Fe^3+^ consistent with Fe_3_O_4_. Nanoparticle growth was controlled via seed-mediated approaches, enabling expansion from 4 nm to 16 nm. This method represented a breakthrough in using Fe(acac)_3_ for synthesizing Fe_3_O_4_ NPs via thermal decomposition and was widely adapted in various studies [77,78,79]. The Asadi group conducted a comprehensive study investigating the evolution of nanoparticle size as a function of solvent volume, precursor amount, and precursor-to-surfactant ratio (Figure 7a–c) [79]. They identified two competing mechanisms governing nucleation and growth: (i) concentration-dependent monomer diffusion and (ii) monomer stabilization by excess surfactant. The former dominates when the solvent volume (and thus, to some extent, the precursor concentration) is varied. At a fixed solvent volume, reducing the precursor amount decreases the precursor-to-surfactant ratio, leading to enhanced monomer stabilization, reduced nucleation, and consequently the growth of larger nanoparticles. Incorporating additional metal precursors such as Co(acac)_2_ and Mn(acac)_2_ further enabled the synthesis of monodisperse spinel ferrites, including CoFe_2_O_4_ and MnFe_2_O_4_, with tunable sizes between 3–20 nm [80].

A major drawback of Sun’s method is the cost of HDD. Rossi et al. investigated alternative mono- and dihydroxy compounds, with or without cyclic structures (e.g., 1,2-octanediol, 1,2-hexanediol, 1,6-hexanediol, 1-hexanol, cyclohexanol, 2-ethylhexanol, 1-octadecanol), showing that HDD could be replaced without significant loss of product quality, though nanoparticle sizes remained <10 nm [78]. García et al. developed a two-step HDD-free synthesis [81]. Fe(acac)_3_, OA, and OAm in BE were heated under inert conditions to produce ~9 nm IONPs. Refluxing the same mixture yielded larger (15 nm) particles, demonstrating tunable size control without expensive reagents.

Parallel efforts focused on biocompatibility and greener syntheses. Gao et al. used 2-pyrrolidone as solvent, yielding hydrophilic IONPs (5–11 nm) with saturation magnetizations of 31 and 65 emu/g [74]. While this method produced IONPs with hydrophilic surfaces suitable for biomedical testing, it did not provide broad size tunability or high saturation magnetization. Theppaleak et al. developed a one-pot method in tetraethylene glycol dimethyl ether with carboxylic acid-terminated poly(ethylene glycol) monomethyl ether (mPEG acid), poly(vinyl alcohol) (PVA), and Jeffamine M-2070, forming a thin polymer shell on IONPs that enhanced colloidal stability [82]. Similarly, Wang et al. designed a phenol-based one-pot pathway, with phenol serving as both reducing agent and ligand, enabling size control through stoichiometry and reaction time [83]. Pinna et al. reported a solvothermal synthesis using benzyl alcohol as both solvent and ligand, requiring lower temperatures (175–200 °C) than conventional ether systems [84]. This approach yielded surfactant-free NPs, though with broader size distributions.

Simplified reaction schemes have also emerged. Xu et al. introduced a two-component system of Fe(acac)_3_ and OAm in BE [71]. In this approach, OAm acted as both stabilizer and reducing agent. By adjusting the OAm/BE ratio, particle sizes between 7 and 10 nm were obtained. This reduction in chemical complexity improved reproducibility and scalability, while producing amine-capped surfaces favorable for ligand exchange. All IONPs exhibited superparamagnetic properties, with saturation magnetization of 76–80 emu/g at 300 K. In contrast, Moya et al. replaced OAm with OA as the sole surfactant in the Fe(acac)_3_/BE system [85]. By varying OA concentration, particle sizes ranging from 7 to 100 nm were achieved. OA played multiple mechanistic roles, including iron oleate formation, growth regulation, and nucleation control. Higher OA concentrations promoted earlier nucleation and smaller sizes, while lower concentrations delayed nucleation, producing larger NPs.

Syntheses of IONPs from Fe(acac)_3_ precursors in different solvent systems and with various types of surfactants have also been reported. Shi et al. developed a synthesis route using ODE as solvent, with OA, trioctylphosphine oxide (TOPO), OAm, and Fe(acac)_3_ [86]_._ The effects of additional surfactants, including sodium dodecyl sulfate (SDS), hexadecyltrimethylammonium bromide (CTAB), PEG, and poly(vinyl pyrrolidone) (PVP), were systematically investigated. TEM analysis revealed that SDS and CTAB promoted aggregation, whereas PEG and PVP yielded monodispersed NPs, indicating that the latter two are more effective stabilizers. Depending on surfactant type and concentration, particle sizes ranged from 9 to 15 nm. A subsequent study further confirmed PEG as an efficient surfactant for biomedical applications such as targeted drug delivery and magnetic hyperthermia in cancer therapy [87]. Orsini et al. employed TOPO as a co-ligand with OA and OAm in Fe(acac)_3_ decomposition, using ODE and 1-eicosene as solvents to investigate the effects of solvent boiling point, alkane chain length, and heating profile on superparamagnetic IONP formation [88].

Oliveira et al. developed a protocol using imidazolium-based ionic liquids (ILs) as both solvent and stabilizing agent, providing a green and recyclable alternative to conventional organic solvents [89]. The method produced superparamagnetic IONPs (8–15 nm) with saturation magnetization values up to 70 emu/g. The favorable performance was attributed to the thermal stability, viscosity, and coordination properties of ILs. In 2017, Wagle et al. employed the IL trihexyltetradecylphosphonium bis(trifluoromethylsulfonyl)imide ([P_6,6,6,14_][Tf_2_N]) as both solvent and capping agent [90]. Long alkyl chains stabilized the NPs without requiring surfactants such as OA or OAm, though a reducing agent was necessary. The method produced uniform superparamagnetic nanocrystals (~14 nm). Importantly, the IL was recyclable for multiple synthesis cycles without compromising nanoparticle quality (Figure 7d). [P_6,6,6,14_][Tf_2_N] remained stable up to 300 °C, but degraded at 350 °C.

**Figure 8:**
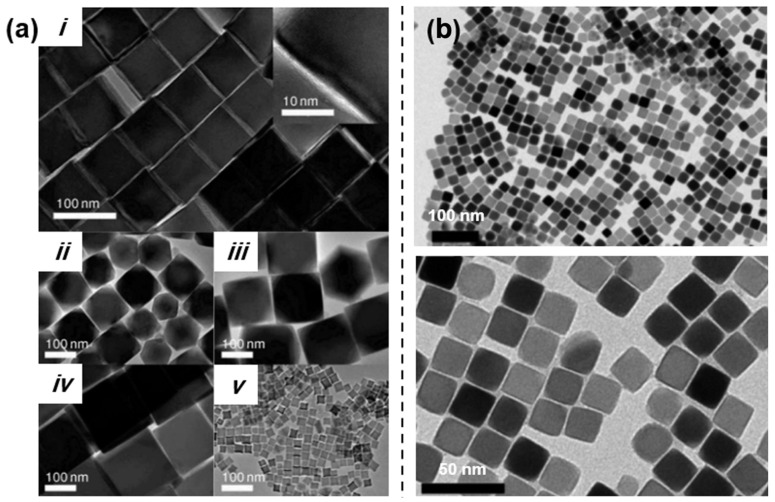
(**a**) TEM images of Fe_3_O_4_ NPs with different morphologies and sizes: (**i**) 79 nm nanocubes, (**ii**) a mixture of truncated cubic and truncated octahedral particles (average 110 nm), (**iii**) 150 nm truncated nanocubes, (**iv**) 160 nm nanocubes, and (**v**) 22 nm nanocubes. Reproduced with permission from Ref. [69]. Copyright 2009 American Chemical Society. (**b**) TEM images of IONPs with cube edge lengths of 19 ± 3 nm. Reproduced with permission from Ref. [93]. Copyright 2012 American Chemical Society.

***Key Precursors for Ferrimagnetic Fe_3_O_4_ Nanocubes.*** Early Fe(acac)_3_-based syntheses primarily produced spherical IONPs < 25 nm. Achieving uniform IONPs > 30 nm remains challenging due to the need to sustain crystal growth into the ferromagnetic size regime, where strong magnetic dipole interactions occur. In 2009, Hyeon’s group developed a method using Fe(acac)_3_, OA, and BE to produce Fe_3_O_4_ nanocubes ranging from 22 to 160 nm (Figure 8a) [69]. Formation of 22 nm nanocubes required addition of 4-biphenylcarboxylic acid, while larger sizes (79–160 nm) were achieved by adjusting Fe(acac)_3_/OA ratios and reaction times. All Fe_3_O_4_ nanocubes exhibited strong ferrimagnetism. Notably, their highly pure magnetite phase and single-crystal nature enabled observation of the predicted single-domain to multi-domain transition in coercivity, and they displayed stronger ferrimagnetic properties than spherical particles of comparable size [91,92]. This work represented a significant milestone, providing reproducible protocols for highly uniform nanocubes and, for the first time, access to NPs within the multi-domain ferromagnetic regime.

Around the same time, a series of studies by Guardia et al. employed decanoic acid for the synthesis of nanocubes, demonstrating remarkable control over nanoparticle size and shape [93,94,95,96]. In 2010, Guardia and co-workers introduced a thermal decomposition method using decanoic acid as the surfactant [94,95]. The rationale was to form fewer stable seeds compared to iron(III) oleate, thereby enabling improved control over nanoparticle growth. By adjusting the precursor-to-ligand ratio, reaction temperature, and heating profile, this approach enabled the synthesis of crystalline IONPs ranging from 5 to 50 nm, with either cubic or spherical morphologies and exhibiting superparamagnetic or ferrimagnetic properties. Nanocubes with sizes between 12 to 38 nm (Figure 8b) were subsequently investigated in detail for magnetic hyperthermia applications [93]. This study offers a comprehensive investigation of the heating performance of NPs from the superparamagnetic to the ferromagnetic regime. Later, Guardia and co-coworkers developed a method to produce both octahedra and nanocubes with edge lengths ranging from 14 to 80 nm for evaluating their potential benefits in magnetic hyperthermia. In this variation, instead of using DBE alone, a mixed solvent system of DBE and squalane (a long-chain alkane) was employed. This strategy allowed fine control over nanoparticle size and morphology through modulation of heating rate and solvent polarity. Subsequently, Guardia et al. reported the use of a binary solvent system of squalene and DBE, combined with controlled heating rates and an optimized decanoic acid-to-Fe(acac)_3_ ratio, to fine-tune nanocube sizes from 14 to 35 nm and octahedral sizes from 40 to 100 nm [96].

**Figure 9:**
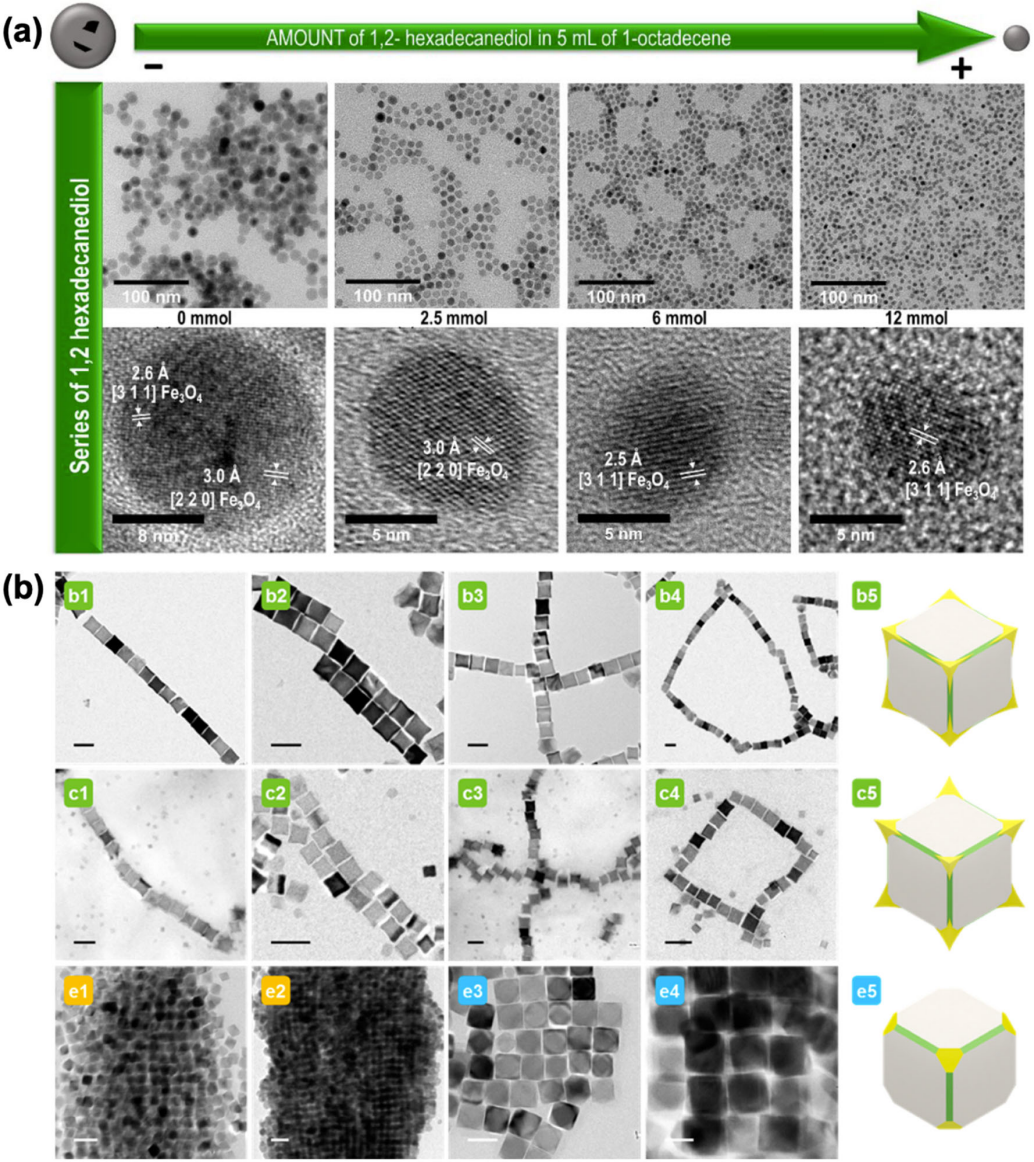
(**a**) TEM images of IONPs synthesized with varying amounts of 1,2-hexadecanediol and ODE, showing the evolution of morphology and crystallinity. Reproduced with permission from Ref. [73]. Copyright 2021 American Chemical Society. (**b**) TEM images of self-assembled magnetite (Fe_3_O_4_) nanocrystals with different morphologies and sizes, forming chains, rings, and clusters driven by magnetic dipolar interactions (scale bars: 50 nm). Reproduced with permission from Ref. [70]. Copyright 2017 American Chemical Society.

***Refining Established Syntheses and Developing Mechanistic Understanding.*** In 2021, Moya et al. examined the influence of HDD and ODE on the morphology, oxidation state, and magnetic properties of IONPs (Figure 9a) [73]. They found that high concentrations of either HDD or ODE produced small, single-phase IONPs with superparamagnetic properties. In contrast, low concentrations of either reagent yielded larger, mixed-phase NPs (Fe_3_O_4_ and FeO) with reduced saturation magnetization due to the presence of the antiferromagnetic FeO phase. HDD promotes nucleation, leading to an increased number of nucleation sites and a reduced concentration of precursor complexes. The subsequent growth process is governed by slow diffusion, resulting in small single-phase IONPs. Hadadian et al. investigated the effects of reagent ratios, showing that variations influenced not only nanoparticle size but also the microstructure of MIONPs [97]. These microstructural changes resulted in significant differences in magnetic properties, even for particles within a narrow size range of 8–13 nm. Qiao et al. attempted to standardize size- and shape-controlled synthesis of Fe_3_O_4_ NPs by systematically examining all key reagents and mapping the role of each component in determining nanoparticle morphology, resulting in an atlas for synthesis control [70]. In this work, the role of the common solvent BE was further elucidated. BE is susceptible to oxidation in air, and its oxidation products strongly influence nanoparticle shape. Controlled experiments were conducted to develop strategies for replacing BE with more stable solvents. Efficient and highly reproducible formulations were designed to synthesize MIONPs ranging from 4 to 55 nm, with morphologies including tetrahedra, octahedra, tetradecahedra, cubes, and stars. The research conducted an in-depth study to evaluate the effects of solvent impurities, ligands, and heating profiles, with the goal of developing a highly reproducible synthetic protocol [70]. Revisiting Sun’s thermal decomposition of Fe(acac)_3_, they discovered that BE impurities—particularly benzaldehyde—played a crucial role. Using aged BE exposed to air, or deliberately bubbling air through fresh BE, consistently produced more monodisperse IONPs. A BE-free system composed of ODE and benzaldehyde also yielded highly monodisperse IONPs with excellent reproducibility. Further experiments revealed that OA promoted rapid nucleation, producing smaller NPs, whereas OAm favored larger particle formation by slowing growth. The choice of diol also influenced morphology: the shorter-chain 1,2-tetradecanediol (TDD) produced smaller NPs compared to HDD. Replacing BE with a less polar solvent mixture of ODE and benzaldehyde resulted in smaller NPs due to reduced monomer solubility and mobility. Heating rate and reaction duration also affected size distribution; a slower heating rate produced smaller particles. By carefully optimizing these parameters, they synthesized IONPs in cubic, spherical, and star morphologies, with sizes ranging from 4 to 55 nm, and achieved precise control over phase and magnetic properties (Figure 9b). This work provides a rational framework for achieving a reliable and tunable thermal decomposition of Fe(acac)_3_, although it remains limited by the requirement for multiple reagents.

Numerous reports have addressed the control of seed growth and nanoparticle size by adjusting parameters such as the concentration of solvent, surfactant, reducing agent, and iron precursor. However, no systematic investigation into the effects of varying reagent quantities was reported until 2017, when the Asadi research group conducted a comprehensive study to address this gap [79]. In their standard synthesis procedure, BE was used as the solvent, OA and OAm as surfactants, and a diol as a reducing agent. The results revealed a non-monotonic correlation between iron precursor and surfactant concentrations. Nanoparticle size increased with higher iron precursor concentrations, either by decreasing the solvent volume or increasing the amount of Fe(acac)_3_, but the trend reversed at very high precursor loads. Although this approach required multiple reagents, it helped reconcile discrepancies among previous studies.

**Figure 10:**
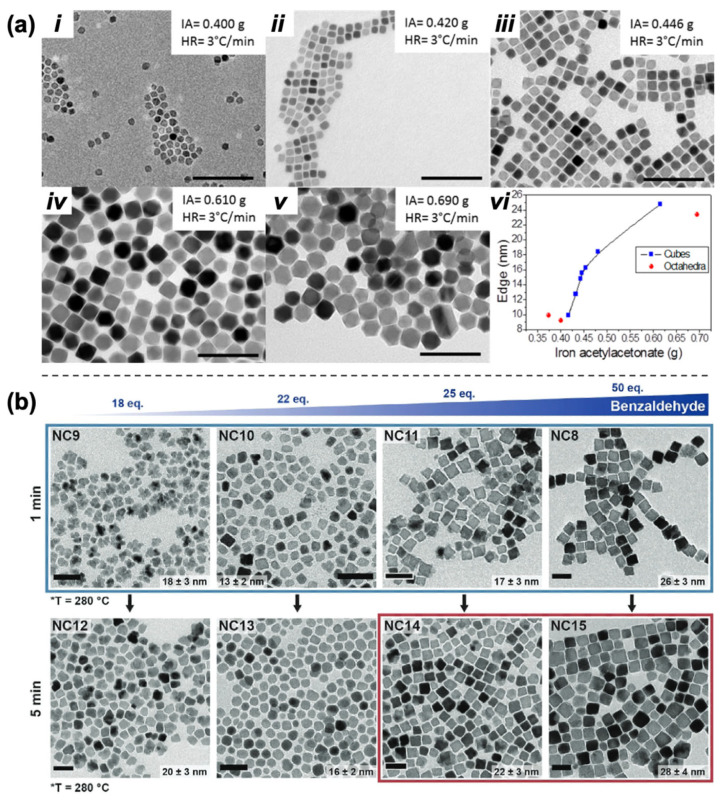
(**a**) TEM images showing the effect of Fe(acac)_3_ concentration on the size and morphology of Fe_3_O_4_ nanocubes, with edge length plotted as a function of Fe(acac)_3_ amount, illustrating size tuning and morphological transitions (scale bars: 100 nm). Reproduced with permission from Ref. [68]. Copyright 2019 American Chemical Society. (**b**) Bright field transmission electron microscopy (BF-TEM) images showing the effects of reaction temperature (250–280 °C) and time (30 s to 5 min) on iron oxide nanocube size and shape (scale bars: 50 nm). Reproduced with permission from Ref. [98]. Copyright 2025 Wiley.

The concentration of Fe(acac)_3_ also significantly influences nucleation kinetics, directly affecting nanoparticle size and morphology [68]. Muro-Cruces et al. demonstrated the synthesis of Fe_3_O_4_ nanocubes with sizes ranging from 9 to 80 nm through systematic variation of Fe(acac)_3_ concentration from 0.4 to 0.61 g (1.12–1.73 mmol), highlighting the precursor’s critical role in controlling nucleation and growth dynamics (Figure 10a). A monotonic increase in nanocube size with increasing Fe(acac)_3_ concentration was observed, while cubic morphology was preserved within a specific concentration range. Morphological control was further enhanced by NaOL, which modulated the monomer chemical potential, enabling the production of nanocubes with narrow size distributions across a wide size range. In this study, the instability of BE solvent during the synthesis was noted as a limitation for maintaining the reaction temperature required for the growth of larger cubic nanocrystals. This limitation was addressed by employing a ternary solvent system composed of BE, ODE, and tetradecane.

The role of benzaldehyde (byproduct of BE decomposition) as a shape-directing agent for nanocube formation was further supported by two recent studies from the Teresa Pellegrino group using both Fe(acac)_3_ and Fe(CO)_5_ precursors [98,99]. In the study employing Fe(acac)_3_, microwave-assisted synthesis—offering significant advantages by reducing reaction time—was introduced. The size of Fe_3_O_4_ nanocubes, ranging from 13 to 30 nm, was tuned by adjusting the amount of benzaldehyde, reaction temperature, and reaction time (Figure 10b) [98].

Under thermal decomposition conditions, a pre-decomposition step at approximately 373–393 K can be introduced, potentially affecting the resultant phase composition (wüstite versus ferrite) and morphology, thereby influencing the magnetic properties of the nanoparticles [100]. While the FeO phase is typically observed in syntheses employing iron(III) oleate, the pre-decomposition step can modify the reaction conditions, leading to the formation of FeO@Fe_3_O_4_ core–shell nanoparticles. The decomposition rate of the precursor, modulated by bulky ligands such as didodecyldimethyl ammonium bromide (DDAB) or tridodecylmethyl ammonium iodide (DDAI), has been shown to govern the formation of multiply twinned structures [101]. These twinned NPs exhibit increased coercivity and altered magnetic domain behavior compared with their non-twinned counterparts.

**Figure 11:**
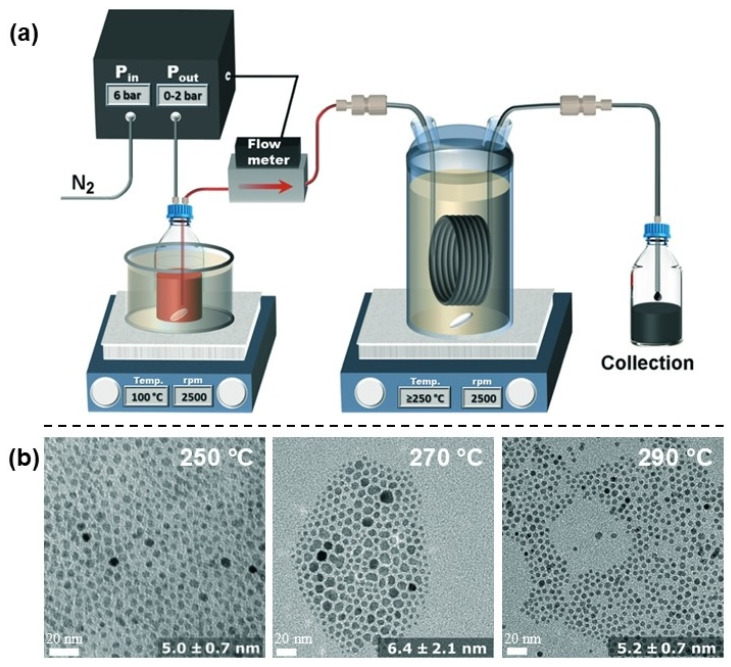
(**a**) Schematic of the continuous thermal decomposition synthesis setup. (**b**) TEM images of IONPs synthesized at 250, 270, and 290 °C. Reproduced with permission from Ref. [104]. Copyright 2020 The Royal Society of Chemistry.

***Improving Scalability by Flow Reactor and Continuous Synthesis.*** In addition to elucidating the role of chemical components, refining synthesis protocols, and investigating mechanistic aspects, recent efforts have emphasized engineering solutions to enable scale-up and achieve larger-scale nanoparticle production. Flow reactor and continuous synthesis approaches have been employed for the production of MIONPs. Gao’s group conducted a comprehensive study on the influence of residence time, fluid velocity, and tubular reactor dimensions on the particle size distribution of PEGylated Fe_3_O_4_ NPs [102]. Uson et al. utilized a continuous polyol-based process to obtain ultrasmall SPIONPs [103]. In a collaborative study, Gavriilidis and Thanh developed a continuous-flow thermal decomposition method in a millifluidic tubular reactor (Figure 11) [104]. This approach employed Fe(acac)_3_ as the precursor, OAm as the surfactant, and ODE as the solvent, while omitting OA to minimize reactor wall adhesion and clogging. Monodisperse IONPs were continuously synthesized within 3–10 min, addressing key challenges in scalability, reproducibility, and size control. However, stabilization using OAm alone is limited due to its weaker binding affinity compared with OA, resulting in polydispersity and susceptibility to oxidation. Overall, although continuous-flow synthesis of MIONPs from Fe(acac)_3_ precursors is still in the early stages of development, this approach demonstrates considerable potential for scalable and reproducible production, representing an important advance toward biomedical applications.

### 2.3. Iron Pentacarbonyl Precursor

***Introduction to Iron Oxide Nanoparticle Synthesis Using Iron Pentacarbonyl.*** Iron pentacarbonyl (Fe(CO)_5_) is widely employed as a precursor for the synthesis of IONPs via thermal decomposition. Upon heating to approximately 100 °C, Fe(CO)_5_ releases carbon monoxide (CO), which, in the presence of appropriate reactants and surfactants, facilitates the nucleation and growth of IONPs [105]. This section outlines the progression of Fe(CO)_5_-based synthesis methods, from early approaches with limited control that produced amorphous materials, to more advanced strategies that enable the preparation of NPs with tunable magnetic properties, morphology, size, and dispersion. The following sections describe how Fe(CO)_5_ has been integrated into different synthetic systems to achieve precise control over nanoparticle characteristics.

***Early Synthetic Approaches and Mechanistic Insights into Iron Pentacarbonyl-Based Thermal Decomposition.*** Two of the earliest reports on the synthesis of crystalline, spherical, and monodisperse maghemite NPs (γ-Fe_2_O_3_) were published by Hyeon and co-workers in 2001 [28]. The procedures included (1) the oxidation of metallic Fe NPs, pre-synthesized via thermal decomposition of Fe(CO)_5_ in the presence of OA and DOE, followed by treatment with trimethylamine *N*-oxide (TMAO) at reflux, and (2) the direct oxidative decomposition of Fe(CO)_5_ in the presence of lauric acid (LA), TMAO, and DOE at reflux. The first approach produced highly crystalline and monodisperse γ-Fe_2_O_3_ nanospheres with particle sizes tunable between 4 and 16 nm by varying the OA concentration. The second method yielded uniform 13 nm nanospheres with high crystallinity. Importantly, the first approach demonstrated superior reproducibility and more reliable control over particle size across different batches, thereby presenting greater potential for scale-up. This comparison highlights the critical role of precursor design and surfactant interactions in achieving controlled nanoparticle synthesis.

**Figure 12:**
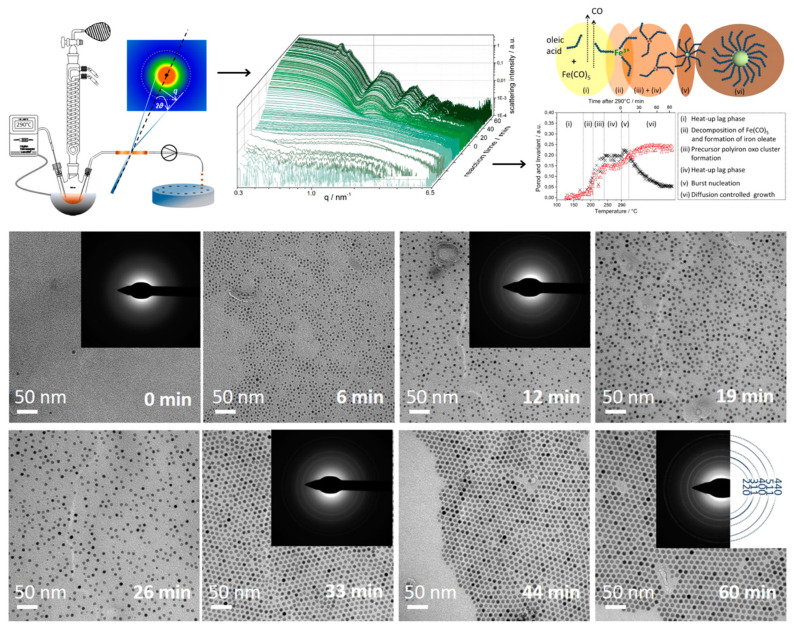
Schematic of the experimental setup; scattering curves of 9–10 nm IONPs showing temporal evolution of the SAXS signal during the reaction; distinct phases identified in IONP synthesis; and TEM images with selected-area electron diffraction (SAED) patterns at different reaction times. Reprinted with permission from Ref. [106]. Copyright 2017 American Chemical Society.

The second approach was later modified by substituting OA for LA, enabling investigation of the effects of the OA/Fe(CO)_5_ molar ratio and heating rate on particle growth kinetics and morphology [106]. In situ small-angle X-ray scattering (SAXS) and wide-angle X-ray scattering (WAXS) were employed to monitor nucleation and growth in real time. Based on these data, the thermal decomposition of Fe(CO)_5_ was found to proceed through six distinct stages (Figure 12): (i) an initial heat-up phase without detectable structural changes; (ii) decomposition of Fe(CO)_5_ to form iron oleate complexes; (iii) formation of precursor clusters, likely polyiron oxo species; (iv) a second lag phase prior to nucleation; (v) burst nucleation triggered by supersaturation; and (vi) particle growth accompanied by size focusing. The final nanoparticle size and dispersity were strongly influenced by both the precursor-to-surfactant ratio and the heating rate, which determined the characteristics of intermediate clusters and the timing of nucleation. This study represents one of the earliest mechanistic investigations of Fe(CO)_5_-based IONP synthesis and provided a framework for minimizing polydispersity and improving scalability.

**Figure 13:**
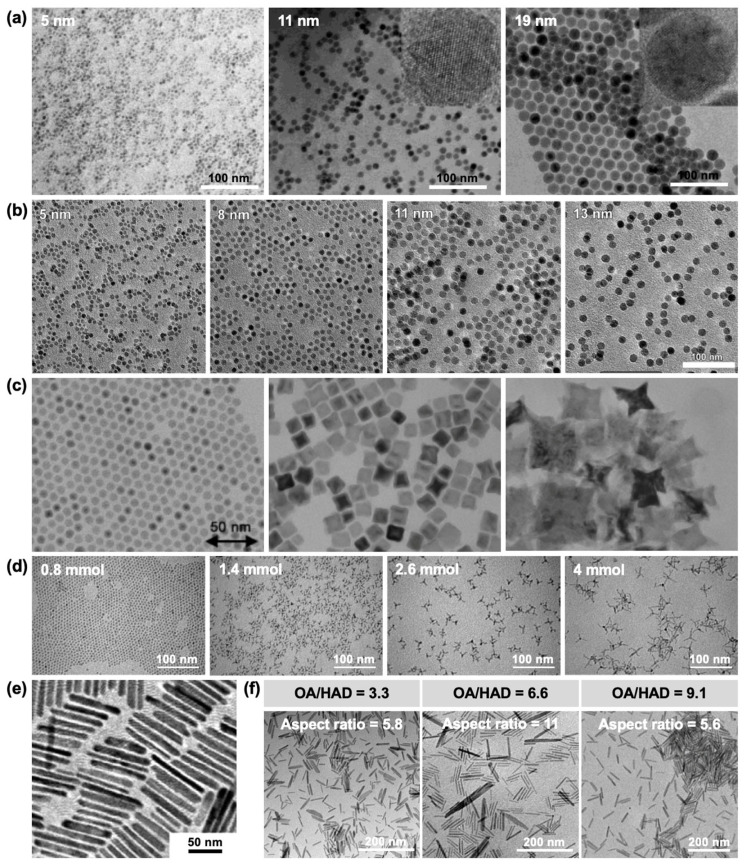
(**a**) TEM images of spherical IONPs with diameters of 5, 11, and 19 nm. Reproduced with permission from Ref. [107]. Copyright 2004 American Chemical Society. (**b**) TEM images of spherical IONPs with average diameters ranging from 5–11 nm. Reproduced with permission from Ref. [108]. Copyright 2006 American Physical Society. (**c**) TEM images of IONPs with spherical, cubic, and star-like morphologies. Reproduced with permission from Ref. [114]. Copyright 2004 American Chemical Society. (**d**) TEM images of IONPs with tetrapod morphology synthesized using Fe(CO)_5_ amounts ranging from 0.8 to 4 mmol. Reproduced with permission from Ref. [115]. Copyright 2006 American Chemical Society. (**e**) TEM images of nanorod-shaped IONPs. Reproduced with permission from Ref. [116]. Copyright 2012 American Chemical Society. (**f**) Effect of the OA-to-HAD ratio on nanorod dimensions. Reproduced with permission from Ref. [117]. Copyright 2016 American Chemical Society.

***Morphological and Size Control of Iron Oxide Nanoparticles* via *Iron Pentacarbonyl-Based Synthesis.*** γ-Fe_2_O_3_ NPs have been synthesized through thermal decomposition of Fe(CO)_5_ under varying OA-to-Fe(CO)_5_ molar ratios [107]. Increasing the OA/Fe(CO)_5_ ratio up to 3:1 promoted the formation of larger NPs, while further increases beyond this ratio produced smaller NPs, consistent with the results reported by Hyeon et al. [28]. Depending on the oxidation conditions, three distinct average sizes—5, 11, and 19 nm—were obtained (Figure 13a). The variation in oxidation state was attributed to the increased surface-to-volume ratio of smaller NPs, which enhanced interactions with O_2_. This, in turn, affected the degree of iron oxidation, producing either pure Fe(III) or mixed Fe(III)/Fe(II) states, and resulted in differences in magnetic behavior. Specifically, 5 nm and 11 nm particles exhibited superparamagnetism, whereas 19 nm particles displayed ferrimagnetic properties. A separate study reported a similar trend, where OA-to-Fe(CO)_5_ ratios of 1:1, 2:1, 3:1, and 4:1 yielded NPs with average diameters of ~5, ~8, ~11, and ~13 nm, respectively (Figure 13b) [108]. However, in contrast to earlier observations [28,107], no reduction in size was observed at ratios above 3:1. These discrepancies suggest that nanoparticle growth mechanisms are strongly dependent on the specific reaction environment.

Beyond its established role as a surfactant, OA has also been reported to perform a dual function in Fe(CO)_5_ decomposition [109]. In addition to acting as a surface-capping ligand that controls particle size and prevents agglomeration, OA was shown to elevate the boiling point of the reaction mixture. This increase in boiling point enabled higher reaction temperatures, thereby favoring the growth of larger NPs. While this dual function was critical in the system, other studies have reported OA serving solely as a surfactant and size-controlling agent [51,110,111]. These approaches successfully produced monodisperse IONPs with tunable sizes, underscoring the versatility of OA under different reaction conditions.

Alternative surfactants and solvent systems have also been employed to further refine particle size and dispersity. For example, OAm was substituted for OA in the thermal decomposition of Fe(CO)_5_ in kerosene under an inert N_2_ atmosphere [112]. Varying OAm concentrations (0, 0.0425, and 0.085 M) revealed that 0.0425 M produced the best control over particle shape and dispersity, producing NPs between 4.8 and 10.9 nm after extended heating. In another approach, a dimethylformamide (DMF)/polyvinylpyrrolidone (PVP) system was used as a solvent–stabilizer combination, producing spherical superparamagnetic IONPs with diameters ranging from 50 to 160 nm [113]. These results highlight the significant influence of surfactant and solvent selection on particle size control and dispersity.

Control over morphology, in addition to particle size, has been achieved by optimizing surfactants, oxidizing agents, and solvent compositions during Fe(CO)_5_ thermolysis. For example, cubic, star-shaped, and spherical IONPs (10–100 nm) were synthesized by injecting pyridine *N*-oxide (PyO) and Fe(CO)_5_ into a mixture of DOE, TOA, and diphenyl ether (DPE) containing LA at 100 °C, followed by post-synthetic exchange of LA with OA [114]. By tuning solvent composition, reaction parameters, and the consumption rates of PyO (oxidizer) and LA (surfactant), morphology and particle size could be precisely controlled (Figure 13c).

As synthetic control improved, more complex nanostructures were targeted. A notable example is the tetrapod-shaped IONP, consisting of a central core with four elongated arms [115]. This unique morphology was achieved by combining OAm and OA, which selectively bind to different crystallographic facets. The arm lengths, ranging from ~3 to ~30 nm, could be tuned by varying the Fe(CO)_5_ precursor concentration (Figure 13d). Magnetic measurements demonstrated that increasing arm length correlated with a transition from superparamagnetic to ferrimagnetic behavior. Nanorod-shaped IONPs have also been synthesized using Fe(CO)_5_ with hexadecylamine (HDA) and OA under solvothermal conditions (Figure 13e). Shape anisotropy endowed nanorods with distinct electrochemical and magnetic properties compared to spherical and plate-like IONPs [116]. Further study demonstrated that the aspect ratio—ratio of length to width—of Fe_3_O_4_ nanorods can be tuned by adjusting the OA/HAD ratio, thereby modulating their specific absorption rate (SAR) (Figure 13f). Nanorods exhibited higher saturation magnetization and SAR values compared to spherical and cubic NPs of comparable volume, indicating their potential for magnetic hyperthermia applications [117].

Structural complexity has also been achieved through the formation of core–shell Fe@Fe_3_O_4_ nanostructures under optimized Fe(CO)_5_ decomposition conditions [118,119,120,121,122,123]. For example, Fe@γ-Fe_2_O_3_ core–shell NPs with diameters of 8, 12, and 14 nm were synthesized and evaluated for magnetic hyperthermia [123]. Over time, however, the Kirkendall effect—caused by interdiffusion and vacancy formation—led to hollowing of the iron core, which diminished magnetic performance. Larger particles retained their core–shell structure for longer periods and exhibited superior magnetic properties and heating efficiency [123]. Collectively, these studies emphasize the tunability of size and morphology in Fe(CO)_5_-derived IONPs, while also highlighting the current challenge of developing scalable and reproducible protocols suitable for industrial production.

***Scalable and Reproducible Protocol for Synthesizing Iron Oxide Nanoparticle from Iron Pentacarbonyl.*** A recently developed protocol provides robust control over nanoparticle size and morphology, enabling gram-scale production of ferrite NPs using Fe(CO)_5_ as the iron precursor [99]. Operating at ~200 °C, which is lower than conventional thermal decomposition methods, this approach avoids complications arising from solvent thermolysis and thermal instability. It also eliminates the need for temperature ramping, thereby reducing both time and energy consumption. Defined molecular additives are employed to direct specific morphologies, such as cubic particles. The procedure is straightforward, requiring neither an inert atmosphere nor vacuum conditions, thereby simplifying the experimental setup. Overall, the method is reproducible, scalable, and consistent with good manufacturing and laboratory practices. The following sections demonstrate its capability to tune the morphology and particle size of IONPs and to extend to other nanoferrites.

The morphology of IONPs is influenced by aromatic and aliphatic aldehyde derivatives [99]. Aldehydes such as 4-methylbenzaldehyde, 4-phenylbenzaldehyde, and 3-methoxybenzaldehyde were used to assess the impact of methyl, phenyl, and methoxy substituents on the benzaldehyde ring. Slight morphological variations were observed: 3-methoxybenzaldehyde and 4-phenylbenzaldehyde produced cubic IONPs with rounded edges, while 4-methylbenzaldehyde yielded well-defined cubic NPs. In contrast, *α*-(2-methylpropylidene)-benzeneacetaldehyde (*α*-BZAD), containing an sp^2^-hybridized carbon bonded to both formyl and phenyl groups, produced highly uniform NPs. The nature and position of substituents (meta or para) likely exert inductive electronic effects that stabilize radical intermediates during solvothermal synthesis, promoting selective binding of aromatic aldehydes to specific nanocrystal facets.

To further examine structure–activity relationships, aldehyde with separated formyl and phenyl groups were employed. 2-Phenylacetaldehyde and 3-phenylpropionaldehyde, in which the groups are separated by one or two sp^3^-hybridized carbons, altered the IONP morphology from cubic to faceted or quasi-spherical [99]. Aliphatic aldehydes, including pentanal, heptanal, and decanal, yielded nearly spherical IONPs with narrow size distributions. The particle diameter was tunable according to the aldehyde used: 15 ± 2 nm with pentanal, 17 ± 2 nm with heptanal, and 18 ± 2 nm with decanal.

Further morphological control was achieved by varying the amine type while maintaining benzaldehyde as the directing agent [99]. Replacing HAD with secondary amines (dioctylamine, didodecylamine) resulted in nearly star-like morphologies. Tertiary amines (trioctylamine, tridodecylamine) and OAm yielded well-defined star-shaped IONPs. These changes are attributed to the influence of amine type on the decomposition kinetics of the Fe(CO)_5_, which in turn determines nanoparticle morphology. The chemical composition of the IONPs could also be tuned by introducing additional metal precursors alongside Fe(CO)_5_ into the optimized solvothermal protocols, resulting in various ferrite NPs such as monodisperse cubic Mn_0.6_Fe_2.4_O_4_ and Zn_0.6_Fe_2.4_O_4_ NPs of varying sizes.

## 3. Nanoparticle Characterization

Significant progress in understanding NPs has been achieved through comprehensive characterization, elucidating their structure, composition, crystallinity, and magnetic properties. The combined application of multiple characterization techniques provides valuable insights into structure–property relationships. For example, magnetic measurements not only probe the magnetic behavior of NPs but also yield indirect information on their composition. Similarly, advanced imaging techniques such as TEM and associated analytical methods provide high-resolution morphological images while revealing crystallographic and compositional features. This section outlines how the integration of diverse characterization methods contributes to a comprehensive understanding of MIONPs. While each method provides a distinct perspective, their complementary nature enables a more complete description of structural and compositional features, thereby improving the interpretation and prediction of magnetic behavior.

Electron microscopy techniques, including TEM and scanning electron microscopy (SEM), are widely used to determine particle size, shape, and uniformity due to their capability for direct imaging at the nanoscale. From such images, particle size distributions can be constructed, allowing determination of average size and polydispersity. For non-spherical particles, such as nanocubes, additional parameters including edge length, body-diagonal dimension, and the cubicity index—describing how closely a nanocube approaches a perfect cubic geometry—have been introduced [68]. Size analysis based on imaging is among the most reliable approaches; however, the time required for histogram construction, the subjectivity of manual particle counting, and the choice of statistical fitting function can introduce variability.

**Figure 14:**
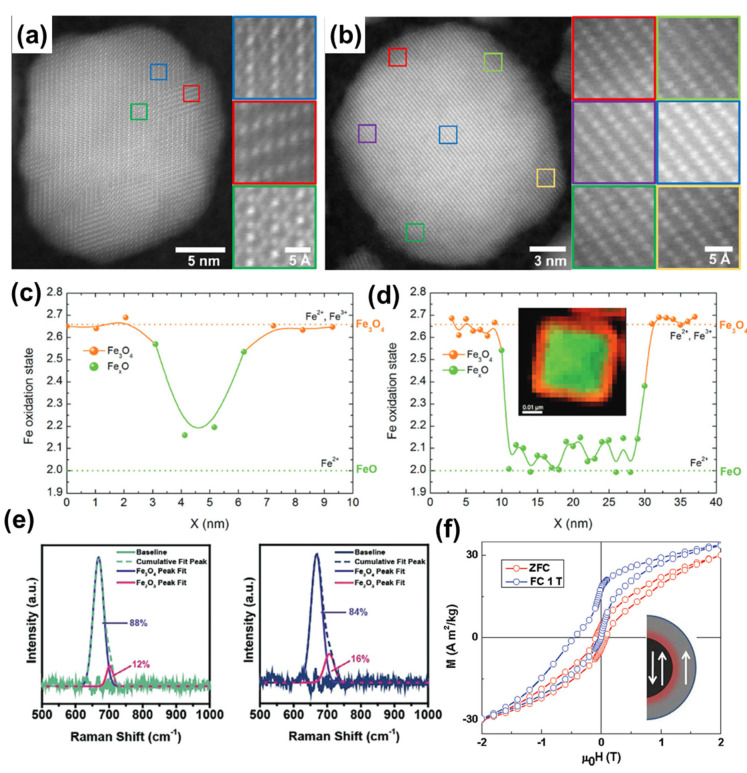
HAADF-STEM images of (**a**) polycrystalline and (**b**) single-crystalline MIONPs. Reproduced with permission from Ref. [6]. Copyright 2017 American Chemical Society. (**c**,**d**) EELS analysis of FeO@Fe_3_O_4_ core–shell NPs. Reproduced with permission from Ref. [124]. (licensed under a Creative Commons Attribution 3.0 Unported License) from Royal Society of Chemistry. (**e**) Raman spectroscopy with spectral deconvolution used to determine the magnetite-to-maghemite composition ratio. Reproduced with permission from Ref. [98]. 2024 Wiley-VCH GmbH (distributed under Creative Commons CC BY license). (**f**) Magnetization versus applied field (M(H)) with Zero-field-cooled/Field-cooled (ZFC/FC) measurements revealing exchange bias interactions in biphasic NPs. Reproduced with permission from Ref. [9]. Copyright 2011 American Chemical Society.

High-resolution scanning transmission electron microscopy (STEM) in high-angle annular dark-field (HAADF) mode enables atomic scale characterization, revealing local defects, strain, tilt, and interfacial boundaries between facets [6,33]. Figure 14a,b illustrates HAADF-STEM images of two samples of similar size but differing crystallinity (polycrystalline versus single-crystal), which strongly influence magnetic behavior. Additionally, electron energy loss spectroscopy (EELS) in TEM provides information on the valence state of Fe cations, thereby offering insights into phase composition [33,51,124]. For example, the FeO@Fe_3_O_4_ core–shell system—commonly obtained via thermal decomposition of iron oleate—can be readily identified using EELS (Figure 14c,d).

Several characterization methods independent of direct imaging have also been applied to nanoparticle size determination. Matrix-assisted laser desorption/ionization time-of-flight (MALDI-TOF) mass spectrometry (MS) has been reported to determine the size of ultrasmall NPs (<5 nm) [42]. This approach has also been extended to quantum dots and plasmonic NPs/nanoclusters [125,126,127]. Dynamic light scattering (DLS) is widely used for determining the hydrodynamic diameter of NPs in suspension [10,36,68]. Many DLS instruments are equipped to measure zeta potential, providing information on surface charge, which is essential for assessing surface chemistry and colloidal stability. The hydrodynamic size typically exceeds the physical size measured by electron microscopy due to solvation effects [36]. For anisotropic morphologies such as nanocubes, the hydrodynamic diameter often corresponds more closely to the body-diagonal than to the edge length, likely reflecting particle rotation in solution [68]. Hydrodynamic size measurements can also be used to assess nanoparticle dispersion, clustering, and aggregation in colloidal form, thereby serving as an important indicator of synthesis quality and colloidal stability.

Small-angle X-ray scattering (SAXS) and small-angle neutron scattering (SANS) provide additional information on the hydrodynamic size and structural organization of NPs [128,129,130]. When combined with DLS, these scattering methods offer complimentary insights into nanoparticle form, stability, and long-range ordering in solution.

Phase analysis or compositional characterization is essential for understanding the material properties of NPs. For MIONPs, identification of the magnetite (Fe_3_O_4_) phase is critical for achieving high magnetic performance; however, detection and quantification of other closely related phases are equally important. Commonly observed phases include γ-Fe_2_O_3_, α-Fe_2_O_3_, FeO, and FeOOH [131]. Among these, the co-existence of γ-Fe_2_O_3_ and Fe_3_O_4_ is most frequently encountered. X-ray diffraction (XRD) can readily distinguish Fe_3_O_4_ from α-Fe_2_O_3_, FeO, and FeOOH, but differentiation between γ-Fe_2_O_3_ and Fe_3_O_4_ is not feasible by XRD alone due to their nearly identical crystal structures [10,132]. XRD can also be used to estimate crystallite size via the Scherrer equation, providing information on crystallinity. In some cases, deconvolution of XRD peaks has been applied to estimate the FeO core size in FeO@Fe_3_O_4_ core–shell NPs [7,130].

Spectroscopic techniques such as X-ray photoelectron spectroscopy (XPS) and Raman spectroscopy are valuable tools for characterizing the phase composition of IONPs. Raman spectroscopy is particularly powerful for differentiating between different iron oxide phases; however, laser irradiation can induce localized overheating and phase transformation of Fe_3_O_4_ to Fe_2_O_3_, leading to inaccurate conclusions. In brief, Raman spectroscopy can reveal the presence of different iron oxide phases, providing a more macroscopic view of composition, but it can be destructive to sensitive samples. Careful control of measurement conditions is therefore necessary. Deconvolution of Raman spectra can further be used to estimate the relative proportions of γ-Fe_2_O_3_ and Fe_3_O_4_ (Figure 14e) [7,98]. XPS analysis also supports phase identification, although it is not conclusive on its own [7,31]. Broadening of Fe 2p peaks indicates the coexistence of Fe^2+^ and Fe^3+^ species, while the absence of satellite peaks near 718 eV supports the presence of highly pure Fe_3_O_4_. Conversely, prominent satellite peaks around 718–719 eV typically indicate impurities such as Fe_2_O_3_. Due to the complexity of Fe 2p spectra, reliable interpretation requires careful peak deconvolution.

In addition to common characterization techniques such as X-ray powder diffraction (XRPD), Raman spectroscopy, and X-ray photoelectron spectroscopy (XPS), several other spectroscopic methods have been employed for the characterization of IONPs. X-ray absorption spectroscopy (XAS) and X-ray magnetic circular dichroism (XMCD) have been used to quantitatively determine the relative proportions of magnetite and maghemite in nanoparticles [27]. Infrared (IR) spectroscopy is also widely used to identify iron oxide phases such as mixture of maghemite and magnetite by focusing on the region from 800 to 400 cm^−1^ [133]. Mössbauer spectroscopy provides detailed information on the local electronic and magnetic environment, enabling differentiation of oxidation states, determination of phase composition, probing of cation distribution, assessment of stoichiometry, and evaluation of magnetic ordering and superparamagnetism. The most commonly used technique is ^57^Mössbauer spectroscopy, which has been shown to be an effective tool for characterizing mixtures of magnetite and maghemite [134]. In addition, the popular FeO@Fe_3_O_4_ core–shell nanoparticle system have been characterized by ^57^Mössbauer spectroscopy at different temperatures to further understand the nano-magnetism and composition of materials [124]. Synchrotron-based X-ray total scattering combined with Debye function analysis has been reported for studying magnetite–maghemite nanoparticles in the 5–15 nm size range [135]. Neutron diffraction and ferromagnetic resonance measurements have been used for characterization of MINOPs [124].

Magnetic measurements, including magnetization versus applied field (*M*(*H*)) and magnetization versus temperature (*M*(*T*)) using the zero-field-cooled/field-cooled (ZFC/FC) protocol, are commonly used to analyze the magnetic properties of MIONPs and can also provide insights into their composition. For example, *M*(*H*) measurements performed at different temperatures under the ZFC/FC protocol can reveal exchange bias interactions arising from the coexistence of different iron oxide phases, illustrated in Figure 14f. Similarly, *M*(*T*) ZFC/FC curves can exhibit characteristic features such as the Verwey transition of crystalline Fe_3_O_4_ at approximately 100–130 K or the Néel transition of FeO at around 200 K [7,10,31]. Therefore, a comprehensive analysis of MIONPs using both *M*(*T*) and *M*(*H*) measurements can yield valuable information on their crystallinity and phase composition.

## 4. Magnetic Properties

The magnetic properties of MIONPs are primarily governed by nanoparticle size and shape [1]; however, recent studies have highlighted the significant roles of phase composition, crystallinity, structural defects, and surface characteristics in determining their behavior. In general, assessing or predicting the magnetic performance of MIONPs cannot be achieved solely by examining particle size and shape. A comprehensive evaluation of structural, compositional, and surface properties at the microscopic level is essential for a complete understanding.

In this section, we first discuss the fundamental principles underlying the magnetic properties of MIONPs, identify key material parameters that influence these properties, and then highlight selected recent discoveries that contribute to a deeper understanding of nanomagnetism.

**Figure 15:**
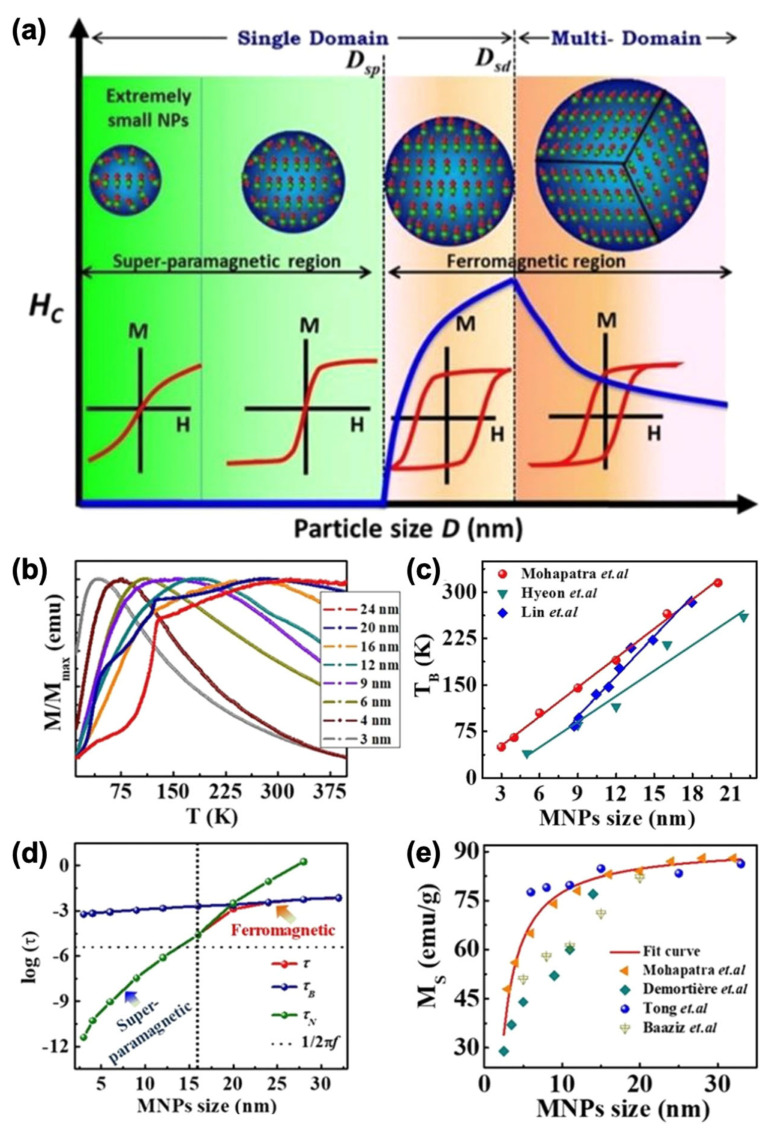
(**a**) Effects of magnetic nanoparticle size on magnetic properties from the superparamagnetic to ferromagnetic regimes. (**b**) Size-dependent zero-field-cooled (ZFC) magnetization curves showing the blocking temperature of MIONPs. (**c**) Relationship between nanoparticle size and blocking temperature. (**d**) Size dependence of Néel and Brownian relaxation times. (**e**) Size dependence of saturation magnetization. Reproduced with permission from Ref. [15]. Copyright 2021 American Chemical Society.

***Fundamental understanding.*** The magnetic properties of nanoparticles are strongly influenced by particle size. For sizes below approximately 5 nm, particles adopt single-domain configurations and exhibit paramagnetic behavior, which is similar to superparamagnetism but characterized by significantly lower saturation magnetization. In this size range, the spin-canting effect dominates due to incomplete alignment of the spins in surface atoms, leading to low saturation magnetization [136]. In the range of 5–25 nm, particles are typically single-domain and superparamagnetic, a regime that has been extensively studied and widely applied in biomedical applications [15]. Around 25 nm—slightly below or above—lies the transition between superparamagnetic and ferromagnetic behavior [1]. Beyond this size, nanoparticles exhibit ferromagnetic properties, with measurable coercivity and remanent magnetization, behaving similarly to permanent magnets. In the ferromagnetic regime, MIONPs remain single-domain up to sizes of roughly 70–150 nm, beyond which they tend to split into multiple magnetic domains. This size transition is denoted as *D*_C_, as described in Equation (1) [15,137], where *A* is the exchange stiffness (J/m), *K*_eff_ is the effective anisotropy constant (in J/m^3^), *µ*_0_ is the vacuum permeability, and *M_S_* is the saturation magnetization. These size thresholds are approximate and may vary depending on particle structure, compositional purity, and crystalline quality. While this description provides a simplified framework for understanding size-dependent magnetic behavior, it is most applicable to ideal cases where particles are uniform, single-crystalline, compositionally pure, and free of defects. Figure 15a illustrates the size effect on magnetic properties, particularly the coercivity parameters [15,138]. In the scope of this paper, most of the nanoparticles discussed are within the superparamagnetic regimes, so coercivity values are negligible when no hysteresis loop is observed.

(1) $$D_{C}\;\approx \;18\frac{\sqrt{AK_{\mathrm{eff}}}}{\mu_{0}{M_{S}}^{2}}$$

(2) $$\tau_{N}=\tau_{0}\;\exp\left[\frac{E_{a}}{k_{B}T}\right]=\tau_{0}\;\exp\left[\frac{K_{\mathrm{eff}}V_{m}}{k_{B}T}\right]$$

(3) $$T_{B}=\frac{K_{\mathrm{eff}}V_{m}}{k_{B}\;\ln(t_{m}/\tau_{0})}$$

(4) $$\tau_{B}=\frac{3\eta V_{H}}{k_{B}T}$$

(5) $$\frac{1}{\tau }=\frac{1}{\tau_{N}}+\frac{1}{\tau_{B}}$$

(6) $$M_{S}=M_{0}\;{\left[1\;-\frac{2d}{D}\right]}^{3}$$

(7) $$K_{\mathrm{eff}}=K_{V}+\frac{6}{D}K_{S}$$

In contrast to the multi-domain particles with multiple magnetization orientations, a single-domain nanoparticle is sufficiently small to be approximated as a superspin with a preferential axis of magnetization, referred to as the easy axis. The superspin can reverse its direction along this axis as “up” and “down” states, separated by an energy barrier *K*_V_. The reversal time can be described by Equation (2), where *E*_a_ is the anisotropy energy (*E*_a_ = *K*_eff_.*V*_m_), *k*_B_*T* is the thermal energy, and τ_0_ is the attempt time (typically 10^−9^ to 10^−13^ s) [15,139]. Here, *K*_eff_ is the effective magnetic anisotropy const, and *V*_m_ is the particle volume. When particle size decreases, the value of *K*_eff_.*V*_m_ can become comparable to, or even smaller than, the thermal energy (*k*_B_*T*). Consequently, the magnetization reversal process driven by thermal energy is influenced by both particle size and temperature. For sufficiently small NPs at high temperatures, the reversal time becomes very short. If the measurement time (t_m_) of magnetic moment is significantly longer than the reversal time, the measured average magnetization approaches zero, corresponding to the superparamagnetic state.

By lowering the temperature, the superparamagnetic behavior of nanoparticle can be blocked. The temperature at which this transition occurs is the blocking temperature (*T*_B_), as described by Equation (3). The blocking temperature (*T*_B_) can be measured from *M*(*T*) ZFC-FC curves and is commonly used to estimate the magnetic anisotropy const [140]. The average *T*_B_ is determined from ZFC measurements as the temperature at which the magnetization curve reaches its maximum. Equation (3) illustrates that *T*_B_ increases with particle size (Figure 15b,c) [15,27,141,142]. Furthermore, the effective anisotropy const can be estimated from *T*_B_ using *T*_B_ = *K*_eff_*V*/25*k*_B_, as ln(τ_m_/τ_0_) is approximately 25 for typical laboratory measurements [15]. For MIONPs, *K*_eff_ is typically in the range of 10 to 20 kJ/m^3^.

In colloidal dispersions, NPs also undergo Brownian motion, with a characteristic Brownian relaxation time given by Equation (4) [21]. This parameter depends on the viscosity of the medium (*η*) and the hydrodynamic size (*V*_H_). The overall effective relaxation time is given by Equation (5) [21,143]. It is important to note that in Equation (4), the volume refers to the hydrodynamic volume, whereas in Equation (3), it refers to the physical volume. Figure 15d illustrates the relationship between τ, τ_N_, and τ_B_ as a function of MIONP (Fe_3_O_4_) size. τ_N_, which reflects magnetic reversal along the easy axis, is more sensitive to particle size, whereas τ_B_ dominates for particles above the superparamagnetic size range [15,141].

The saturation magnetization (*M_S_*) is generally influenced by the particle size. Smaller particles have a higher surface-to-volume ratio, which increases the relative contribution of surface spins to the overall *M_S_* value. In most cases, surface spin disorder or spin canting reduces the total *M_S_*; therefore, smaller IONPs tend to exhibit lower *M_S_* values (Figure 15e) [15,133,144,145]. Equation (6) describes the relationship between *M_S_* and particle size based on a core–shell model, where magnetic core is surrounded by a magnetic disordered shell. In this equation, *M*_0_ is the saturation magnetization of the bulk material, *d* is the thickness of the disordered shell, and *D* is the particle size [20,146].

However, the *M_S_* can be affected by multiple factors. If nanoparticles possess a highly crystalline magnetite phase with minimal defects and negligible surface disorders, IONPs can achieve *M_S_* values close to the bulk value (~90 emu/g), even at small particle sizes. Surface anisotropy (*K*_S_) is an important contributor to the effective anisotropy (*K*_eff_), which governs the magnetic properties of NPs. Equation (7) relates *K*_S_ to *K*_eff_, volume anisotropy, and particle size. For example, surface anisotropy of nanoparticles with different shapes can be calculated using Equation (7) [11,140]. These considerations underscore the critical influence of structural and surface properties on the magnetic behavior of nanoparticles.

***Beyond the Size Impact—Important Role of Crystallographic Defects and Surface.*** Recent studies have revealed numerous factors that govern the properties of nanoparticles. Therefore, to understand the nano-magnetism of these particles, correlations between size and shape alone are insufficient; examination at more microscopic levels of nanoparticle structure is essential. Nanoparticles with similar size and shape may exhibit different magnetic properties due to variations in composition purity and crystallographic characteristics at the microscopic scale. Consequently, a comprehensive understanding of their magnetic properties requires advanced materials characterization and the integration of complementary characterization techniques.

**Figure 16:**
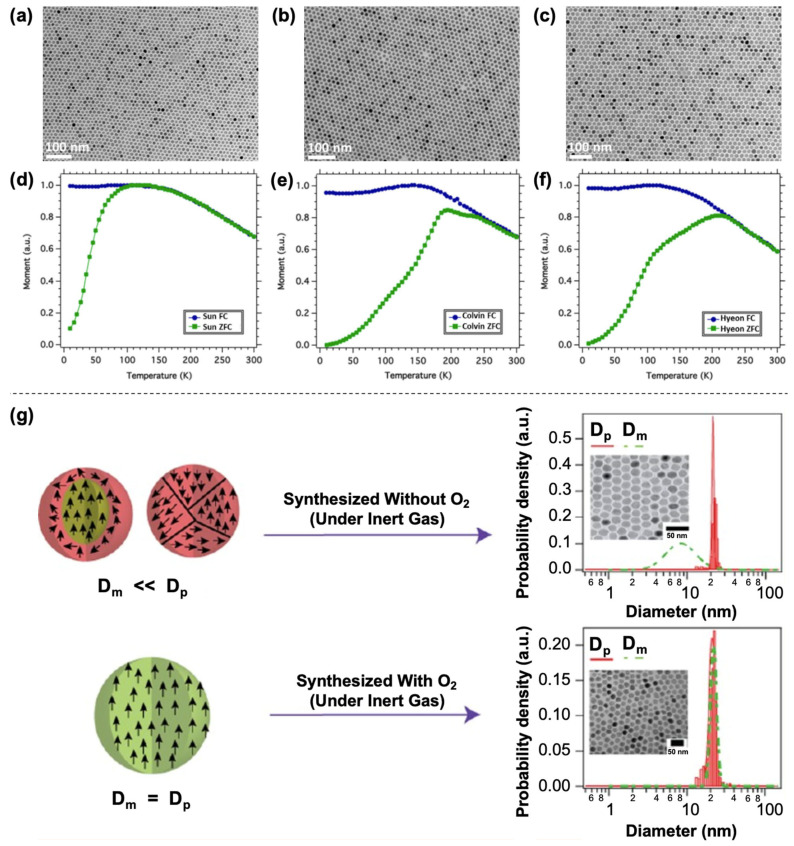
(**a**–**f**) MIONPs of similar size exhibiting different magnetic properties determined by the crystallographic characteristics. Reproduced with permission from Ref. [13]. (distributed under Creative Commons CC BY License). (**g**) Illustration of magnetic diameter versus physical diameter for optimizing the magnetic properties of MIONPs. Reproduced with permission from Ref. [6]. Copyright 2017 American Chemical Society.

Nedelkoski et al. studied IONPs in the size range of 12 to 14 nm from the Sun (size 12.3 ± 2.9 nm) [80], Colvin (size 13.7 ± 1.6 nm) [147], and Hyeon (size 14.2 ± 2.0 nm) groups [27], and reported important insights into the defects and phase boundaries of typical single-domain NPs [13]. The samples, synthesized by leading research groups in the field, exhibited comparable size and uniformity but differed in saturation magnetization values and displayed distinct features in the *M*(*T*) ZFC/FC curves (Figure 16a–f). The M_S_ values for the sample from Sun’s group was 71 emu/g (at 300 K), whereas samples from the other groups showed lower *M_S_* values in the range of 36–40 emu/g. Atomically resolved HAADF-STEM images revealed that Sun’s nanoparticles exhibited no strain and disorder across the particle. In contrast, evidence of defects was observed in samples from the other groups. These defects formed antiphase boundaries, and the strong antiferromagnetic interactions across these boundaries support the presence of multiple magnetic domains even at such small nanoparticle sizes.

Unni et al. refined the synthesis of NPs to improve saturation magnetization and used the magnetic dead layer concept to account for the difference between the physical diameter and the magnetic diameter of NPs (Figure 16g) [6]. The magnetic diameter has often been neglected, with the physical diameter typically assumed to represent the entire magnetic volume. However, surface disorder and spin canting effects can create a shell of a magnetic dead layer. The researchers optimized the synthesis to minimize this magnetic dead layer, thereby producing NPs in which the magnetic diameter closely matched the physical diameter, maximizing their magnetic properties.

***Magnetic Anisotropy and Understanding the Magnetism of Individual Nanoparticles.*** Recently, advancements in the understanding of nanomagnetism have enabled the quantification of the magnetic properties of nanoparticles at the individual level. Conventional magnetic measurements generally characterize an entire population of nanoparticles, in which significant interparticle interactions may exist. As a result, the measured magnetic properties often reflect the collective behavior of the population rather than those of individual nanoparticles. To address this, researchers introduced non-magnetic SiO_2_ shell coatings to spatially separate nanoparticles, thereby minimizing interparticle interactions and allowing closer examination of individual nanoparticle properties [11,12].

The study by García-Acevedo et al. was conducted on various iron oxide cores coated with silica shells of different thicknesses [12]. The *K*_eff_ values for core sizes of 8.1, 10.2, and 15.3 nm were determined to be 48, 23, and 11 kJ/m^3^, respectively, and were found to be independent of the spatial separation resulting from variations in silica shell thickness. Andersson et al. investigated maghemite nanoparticles by comparing dense particle assemblies with non-interacting particles coated with a silica shell [148]. The results indicated that superexchange is the dominant interparticle interaction in the assemblies, with no significant evidence of surface spin disorder.

The primary objective of these nanomagnetism studies on interaction-free magnetite nanoparticles (MINOPs) is to determine the magnetic anisotropy constant of individual particles, a key parameter for optimizing performance in biomedical applications. A recent study by the Balachandran group revealed counterintuitive insights into the relationships between size, shape, and magnetic anisotropy [11]. Ideal interaction-free magnetite nanoparticles with spherical, cubic, and octahedral morphologies were systematically investigated. The effective anisotropy constant (*K*_eff_) was determined to range from 10–20 kJ/m^3^, with negligible dependence on particle size. This finding contradicts the commonly predicted inverse relationship between *K*_eff_ and particle size expected from surface magnetic anisotropy considerations.

Furthermore, the observed trend *K*_eff_ (octahedral) > *K*_eff_ (spheres) > *K*_eff_ (cubes) contradicts the typical expectation that nanocubes exhibit superior magnetic properties and enhanced magnetic hyperthermia performance. Theoretical analysis indicated that the dominant contribution to the effective anisotropy originates from uniaxial crystalline anisotropy, rather than from shape or surface anisotropy. Additionally, the differences in anisotropy constants among shapes could not be directly explained by the crystalline magnetic anisotropy of the magnetite phase. These findings highlight the need for comprehensive investigations involving nanoparticles with diverse sizes and shapes to fully elucidate the origins of magnetic anisotropy.

## 5. Aqueous Phase Transfer of Hydrophobic Iron Oxide Nanoparticles

IONPs synthesized by thermal decomposition, regardless of the types of iron precursor used, are typically coated with OA or OAm to ensure good dispersibility in non-polar organic solvents. However, most applications, particularly those in biological fields, require IONPs to be dispersed in aqueous media [149]. For in vivo and biosensing applications, surface chemistry must be tailored to provide water dispersibility, colloidal stability in physiological conditions, low nonspecific adsorption, and functional handles for bioconjugation.

***Ligand exchange.*** A common approach is the post-synthetic replacement of native hydrophobic ligands with hydrophilic, multifunctional molecules. Small-molecule ligands bearing catechol/diol anchors (dopamine, DOPA-PEG) bind strongly to the iron oxide surface via bidentate coordination, replacing OA and introducing water solubility. Carboxylate-, phosphate-, and silane-based ligands also form robust coordination layers; phosphonates in particular show high stability across a broad pH range. Ligand exchange can be performed in biphasic systems (organic/water) with surfactant stripping agents (e.g., tetramethylammonium hydroxide) [10,117], mercaptosuccinic acid (MSA) [150], and 2,3-dimercaptosuccinic acid (DMSA) [97].

***Encapsulation strategies.*** Instead of exchanging the ligand monolayer, hydrophobic SPIONs can be encapsulated in amphiphilic polymers or lipid micelles, where the hydrophobic tail intercalates with OA/OAm, while the hydrophilic head provides aqueous dispersibility. Examples include polysorbate-80 [34], poly(maleic anhydride-*alt*-1-octadecene) modified with PEG (PMAO−PEG) [52,99], PMAO [10,68,93], dendron molecules with bi-phosphonate anchors and PEG chains [5], gallol-PEG (GA-PEG) [10,98,99,151], Pluronic F127 co-polymer [110]. This approach preserves the native hydrophobic layer, maintaining core crystallinity and magnetization, but increases hydrodynamic size.

***Zwitterionic and antifouling coatings.*** For long circulation times and reduced protein corona formation, zwitterionic ligands (sulfobetaines and carboxybetaines) and dense PEG brushes are widely used [152,153]. These coatings resist opsonization and nonspecific adsorption, enhancing pharmacokinetics. Recent studies report catechol-anchored sulfobetaine ligands providing months-long stability in serum-containing buffers.

***Reverse microemulsion based phase transfer.*** The reverse microemulsion method offers a powerful approach for simultaneous phase transfer and surface functionalization of hydrophobic IONPs. In this technique, nanoparticles are dispersed in the oil phase of a water-in-oil (*W*/*O*) microemulsion stabilized by surfactants (e.g., CTAB, IGEPAL, or nonionic surfactants), and aqueous reactants are introduced as nanosized droplets [154,155]. These droplets act as nanoreactors, enabling controlled ligand exchange or surface coating reactions (e.g., silica, PEG, or zwitterionic shells) at the oil–water interface. This method provides fine control over shell thickness and composition, good colloidal stability due to sterically or electrostatically stabilized interfaces, compatibility with silica coating and in situ PEGylation. Notably, this route enables direct transfer of IONPs from nonpolar to aqueous phase while simultaneously imparting hydrophilic and functional groups, making it highly suitable for biomedical applications.

***Silica coating and silane chemistry.*** Hydrophobic MIONPs of various sizes and shapes can be readily coated with a controllable SiO_2_ shell thickness using reverse micelle methods [11,12,156]. Although the silica shell reduces saturation magnetization due to its non-magnetic nature, it also decreases interparticle magnetic interactions, thereby minimizing magnetically induced clustering. The silica layer is hydrophilic and rich in surface hydroxyl groups, making it highly versatile for further functionalization via silane chemistry. Functional groups such as amine (–NH_2_) [157,158], carboxylic acid (–COOH) [159,160], and thiol (–SH) [161,162] can be grafted onto the silica surface by introducing the corresponding silane compounds and refluxing under mild conditions. Numerous successful bioconjugations of important biomolecules on MIONPs have been achieved through silica shells, demonstrating the robustness of this approach [158,162,163,164,165,166,167].

***Colloidal Stability of Phase-Transferred IONPs in Physiological Media***. The choice and optimization of the transfer method are critical for achieving high colloidal stability in aqueous environments. These transfer strategies underscore the importance of surfactant and surface chemistry selection—not only during synthesis but also in post-synthetic modification—linking molecular design with real-world application. Beyond initial aqueous transfer, the stability of functionalized IONPs must be validated in physiological environments. For example, DMSA-coated NPs show good dispersibility in PBS but limited stability in serum-containing DMEM [168,169], while catechol-phosphonate PEG ligands remain colloidally stable for weeks [170,171]. Zwitterionic coatings, such as sulfobetaine-anchored catechols, demonstrate exceptional resistance to aggregation and protein adsorption, ensuring prolonged stability in serum [153,172]. Such comparative evaluations underscore that successful aqueous transfer strategies must be benchmarked not only by solubility but also by performance in physiologically relevant buffers and media.

## 6. Conclusions and Outlook

Since the early 2000s, there have been significant developments in materials science and nanochemistry, along with advancements in materials characterization techniques and in situ analysis. These progressions have synergistically contributed to a better understanding of nanoparticle formation mechanisms, refinement of synthetic protocols, and improvements in the reproducibility and scalability of MIONPs. Furthermore, advances in characterization methods have enabled the investigation of material structures at the microscopic level. Concurrently, increased knowledge in nanomagnetism has provided substantial insights into the structure–property relationships of magnetic nanoparticles. Surface functionalization strategies have facilitated the transfer of hydrophobic nanoparticles into various hydrophilic forms with tunable surface functionalities. The use of iron oleate, iron(III) acetylacetonate, and iron pentacarbonyl in thermal decomposition reactions has proven to be among the most effective approaches for synthesizing nanoparticles with homogeneous morphology and potentially high saturation magnetization. Each chemical reagent and technical parameter involved in the synthesis process—such as heating rate, stirring speed, degassing conditions, and experimental setup—has been systematically studied to gain further insights and optimize the synthetic procedure. Although scalability poses engineering challenges due to the complexity of high-temperature synthesis, initial efforts indicate promising potential for scale-up in larger-scale applications. In parallel, ongoing progress in related fields such as chemistry, biology, oncology, and diagnostic and therapeutic technologies is likely to support the integration of efficient MIONPs in biomedical applications, including sensing, MRI, magnetic hyperthermia, and drug delivery. These developments collectively position MIONPs as key components in emerging diagnostic and therapeutic strategies for addressing critical healthcare challenges.

The future design of MIONPs for biomedical applications should be guided by several key principles. First, particles should be engineered to possess appropriate sizes and magnetic properties tailored to the intended application. Second, achieving high uniformity in size and shape will be essential to ensure reproducible performance across batches. Third, minimizing crystallographic defects and surface disorders will be critical to maximize magnetic performance. The synthesis methods used to produce the nanoparticles should aim to satisfy these three principles with high reproducibility and scalability. Finally, surface functionalities must be rationally designed to provide biocompatibility and colloidal stability under physiological conditions. Similarly, research efforts aimed at improving the robustness of surface functionalization methods—with high reproducibility, scalability, and precise control over the density of functionalities—are strongly encouraged.

## Data Availability

No new data were created or analyzed in this study.
